# Qualitative, Quantitative, In Vitro Antioxidant Activity and Chemical Profiling of *Leptadenia pyrotechnica* (Forssk.) Decne Using Advanced Analytical Techniques

**DOI:** 10.3390/antiox13070794

**Published:** 2024-06-28

**Authors:** Divya Kumari, Devendra Singh, Mukesh Meena, Pracheta Janmeda, Manzer H. Siddiqui

**Affiliations:** 1 Department of Bioscience and Biotechnology, Banasthali Vidyapith, Tonk 304022, Rajasthan, India; divyakumari4193@gmail.com; 2 Department of Chemistry, Mohanlal Sukhadia University, Udaipur 313001, Rajasthan, India; dsingh@mlsu.ac.in; 3 Laboratory of Phytopathology and Microbial Biotechnology, Department of Botany, Mohanlal Sukhadia University, Udaipur 313001, Rajasthan, India; mukeshmeenamlsu@gmail.com; 4 Department of Botany and Microbiology, College of Science, King Saud University, Riyadh 11451, Saudi Arabia; mhsiddiqui@ksu.edu.sa

**Keywords:** alkaloids, antioxidant property, *Leptadenia pyrotechnica*, metal chelation, phenols, saponins, tannins

## Abstract

*Leptadenia pyrotechnica* Forssk. Decne (LP) is a medicinal herb from the Asclepiadaceae family with many advantageous properties. The goal of this research is to identify, quantify, and evaluate the antioxidant potential of LP to validate its remarkable therapeutic advantages. The hot soxhlet extraction method was employed to prepare different extracts of LP (stem and root). These extracts were evaluated physiochemically to check their impurity, purity, and quality; qualitatively to detect different phytochemicals; and quantitatively for phenol, saponin, tannin, flavonoid, and alkaloid contents. Then, the in vitro antioxidant potential was estimated by DPPH, NO, H_2_O_2_ scavenging assays, and MC and FRAP assays. The most prevalent phytochemicals of LP were then analysed by AAS, FT-IR, UV–visible, and GC-MS techniques. A higher extractive yield was shown by LPSE and LPRE (7.37 ± 0.11 and 5.70 ± 0.02). The LP stem showed better physicochemical and qualitative results than the root. The quantitative and in vitro antioxidant results indicated maximal phenols, tannins, and alkaloid contents in LPSE, which was further confirmed by UV–visible, FT-IR, and GC-MS results. The extraction methods (soxhlation or ultrasonication) were optimized by utilizing RSM to determine the impacts of multiple parameters. The study concluded that the plant has remarkable therapeutic advantages to promote additional clinical investigations and the mechanisms of its action.

## 1. Introduction

The region of the Indian Himalayas has excited the curiosity of scientists and offers an endless array of different medicinal herbs [1]. Medicinal herbs have been taken advantage of for their therapeutic properties since the start of human history. Therapeutic plants have a prolonged record, which is continuously utilized to create natural medications for a range of illnesses and diseases worldwide [2,3,4,5]. In accordance with the World Health Organization (WHO), approximately 80% of the global human population relies on traditional medical treatments for basic well-being due to the absence of contemporary medicines and economic hardship [6]. With the progress of new pharmaceuticals, the utilization of plants or organic components in medicinal perspectives for a broad range of factors has been boosted worldwide. Due to their harmless nature and fewer recognized negative effects than contemporary generic pharmaceuticals, herbal remedies have often been used in remote regions [7,8].

*Leptadenia pyrotechnica* Forssk. Decne (LP) is a medicinal herb from the *Leptadenia* genus and Asclepiadaceae family with many advantageous properties. It is a massively branched, most often upright, leafless shrub that flourishes in hot, sandy, and arid regions instead of deep, non-saline sand [9]. It stretches throughout the regions of North Africa, the Arabian Peninsula, and Western India from the Northern Sahel region. In addition to Pakistan and India, LP has been identified in Mauritania, Eritrea, Egypt, the United Arab Emirates, Somalia, Saudi Arabia, Qatar, and Bahrain. Some common names for it are Blazing Bush, Desert Broom, Kheep, Khip, and Khimp. According to relevant research, this plant exhibits a broad spectrum of notable medicinal properties, comprising cytotoxic, antibacterial, wound healing, anticancer, antioxidant, antidiabetic, antilipoxygenase, anthelmintic, and antiatherosclerotic properties [10]. Precious phytochemicals such as phenolic acids, flavonoids, cardiac glycosides, terpenes, pregnane glycosides, sugars, alkaloids, fatty acids, amino acids, hydrocarbons, and sterols have been reported to be prevalent in LP [11].

This plant is regarded as a promising natural cure based on prior research, which may be important in the discovery of possible new medications. The data on various parts of the plant (LP) and its biological functions are currently insufficient, even though there have been some studies on phytocompounds and their total content. Therefore, the purpose of this study was to determine the phenolic, saponin, tannin, flavonoid, and alkaloid contents and their antioxidant activities in distinct extracts of LP stem and root using several quantitative assays such as total phenolic content (TPC), total saponin content (TSC), total tannin content (TTC), total flavonoid content (TFC), total alkaloid content (TAC), and in vitro antioxidant activities such as 2,2-diphenyl-1-picryl-hydrazyl-hydrate (DPPH), nitric oxide (NO), hydrogen peroxide (H_2_O_2_) scavenging assays and ferric reducing antioxidant power (FRAP) assays, and metal chelation (MC) assays. The present study also intended to describe the bioactive components in the plant extracts using different spectroscopic methods [atomic absorption spectrophotometer (AAS), ultraviolet–visible (UV–vis), Fourier-transform infrared spectroscopy (FT-IR), and gas chromatography–mass spectrometry (GC-MS)] along with the estimation of their bioactivities. This study also aims to optimize the extraction methods of tannins, phenols, and alkaloids from LP stem using conventional soxhlet extraction (CSE) and non-conventional ultrasound-assisted extraction (NCUSAE), taking ethanol as a solvent. To the best of our knowledge, these techniques utilizing ethanol have not been mentioned earlier and this technology can facilitate mass transfer processes and lower energy usage.

## 2. Materials and Methods

### 2.1. Chemicals and Drugs Utilized

Ascorbic acid (AA), 2,2-Diphenyl-1-picrylhydrazyl (DPPH), gallic acid (GA), trichloroacetic acid (TCAA), quillaja (QJ), rutin (RUT), caffeine (Cf), ferrozine, Folin–Ciocalteu reagent, Folin–Denis reagent, ferric chloride (FeCl_3_), aluminium chloride (AlCl_3_), sodium carbonate (Na_2_CO_3_), sodium hydroxide (NaOH), sodium nitrite (NaNO_2_), tannic acid (TA), vanillin, sulfuric acid (H_2_SO_4_), ethylenediaminetetraacetic acid (EDTA), bromothymol blue (BTB), butylated hydroxytoluene (BHT), hydrochloric acid (HCl), 2,4,6-tri (2-pyridyl)-S-triazine (TPTZ), hydrogen peroxide (H_2_O_2_), sodium nitroprusside, Griess’ reagent, disodium hydrogen phosphate (Na_2_HPO_4_), sodium dihydrogen phosphate (NaH_2_PO_4_), nitric acid (HNO_3_), perchloric acid (HClO_4_), sulfanilic acid (H_3_NC_6_H_4_SO_3_), napthylethylene diamine dihydrochloride (C_12_H_16_Cl_2_N_2_), sodium sulphate (Na_2_SO_4_), and other chemicals and solvents (analytical grade) were purchased from Sigma Aldrich (Burlington, MA, USA) and Hi-Media brands (Maharashtra, India).

### 2.2. Collection of Experimental Plant Material

The experimental plant was collected in the month of January, identified (Altitude: 26°24′14.8414″; Latitude: 73°52′9.7194″), and authenticated by the botanist from Krishi Vigyan Kendra, Banasthali Vidyapith, Rajasthan (304022). A voucher number (BURI-1727/2023) was received, and this species name was submitted to the International Plant Names Index and first published in Annales des Sciences Naturelles, Botanique [12].

### 2.3. Extract Preparation of the Plant Sample

For the current study, only the relevant parts of LP were utilized, namely the stems and the roots. The plant samples were first cleaned using distilled water, allowed to air-dry (15 to 25 days) in the shade, and then roughly crushed with the grinding machine to turn them into fine powder. We used 400 mL each of petroleum ether (PE) (at 60 °C), hexane (H) (at 68 °C), chloroform (CH) (at 61.2 °C), ethyl acetate (EA) (at 77.2 °C), ethanol (E) (at 82 °C), and aqueous (AQ) solvents (at 100 °C) for 8–24 h to prepare six total separate plant extracts. To quantify the secondary metabolites, extracts were dried, stored in desiccators, and then afterwards kept at 4 °C in an airtight apparatus. The extract concentration employed throughout the experiments was 1 mg/mL. The hot soxhlet extraction method was employed for preparing different LP extracts by utilizing 50 g powdered plant material and 400 mL of each of the solvents, which were further kept at 4 °C in an airtight apparatus for further analysis [13]. The percentage of extraction yield for the various LP extracts was calculated by the following formula [14]:

Extract yield (%) = (Weight of sample extract)/(Weight of dry plant powder) × 100

### 2.4. Proximate Analyses

Physicochemical parameters such as the ash content (water-soluble ash, total ash, and acid-insoluble ash), foreign organic matter, crude fibre, crude fat, loss of dryness (LOD), and pH (at 1% and 10%) were determined by utilizing fresh plant samples (powdered forms of stem and root) for the evaluation of impurity, purity, and quality of the crude drugs present in the samples. All the evaluations were carried out according to the WHO guidelines on quality control techniques for medicinal plant materials [15].

### 2.5. Qualitative Screening

Qualitative screening can reveal the presence or absence of numerous kinds of secondary metabolites in a plant. It is based on changes in colour, precipitation, or other macroscopically visible variations. Various primary and secondary plant metabolites, such as carbohydrates, amino acids, flavonoids, tannins, alkaloids, phenols, and saponins, were evaluated in both the stem and root parts of LP extracts.

### 2.6. Quantitative Analysis of Phytocompounds

#### 2.6.1. Total Phenolic Estimation

To 2.5 mL of Na_2_CO_3_ (20%) and 500 µL of Folin–Ciocalteu reagent (1:1 with distilled water) was added 100 µL of sample extract. The solution was properly mixed before being incubated in the dark for 40 min to produce its specific colour. After that, the absorbance was measured at 725 nm following incubation. A calibration curve for the standard gallic acid (GA) was made, and linearity was observed between 100 and 500 µg/mL. The TPC was represented as GA equivalent per 50 g of powdered plant material (µg GA/50 g powdered plant material), using the formula given below [16]:

C = c × V/m

where C = total plant extract content (µg/g); c = concentration determined from standard curve (y); V = extract volume (mL); and m = weight of pure sample extract (g).

#### 2.6.2. Total Saponin Estimation

After mixing 2.5 mL of 72% H_2_SO_4_, 0.25 mL of 8% vanillin (*w*/*v*) in methanol was then added to 0.1 mL of the appropriately diluted LP stem and root extracts. The solution tubes were then sealed, vortexed, and incubated for an additional 15 min at 60 °C, and their absorbance was determined at 560 nm. A standard graph of quillaja (QJ) was prepared and the TSC was represented as QJ equivalents per 50 g of powdered plant material (µg QJ/50 g powdered plant material) [17].

#### 2.6.3. Total Tannin Estimation

The 0.5 mL sample extract was mixed with methanol (0.5 mL), Folin–Denis (0.5 mL) reagent, and 1 mL Na_2_CO_3_ solution (35%). The reaction mixture was mixed with distilled water to make up the volume of 10 mL. The solution was then mixed accurately before being kept for 30 min at room temperature, and the absorbance of the resultant solution was determined at 700 nm. A standard graph of tannic acid (TA) was prepared and the TTC was represented as TA equivalents per 50 g of powdered plant material (µg TA/50 g powdered plant material) [18].

#### 2.6.4. Total Flavonoid Estimation

About 0.1 mL of sample extract was dissolved in distilled water (200 mL) before mixing with 150 mL of NaNO_2_ (5% *w*/*v*) solution. After incubation for five minutes, 150 µL of AlCl_3_ solution (10% *w*/*v*) was added to it, and the mixture was kept for incubation for 6 min. Then, 2 mL of NaOH (4% *w*/*v*) solution was added and made up the volume of the solution with distilled water to 5 mL. After thoroughly shaking, the mixture was kept at room temperature (RT) for 15 min and the absorbance was measured at 510 nm. A standard graph of rutin (RUT) was prepared and the TFC was represented as RUT equivalents per 50 g of powdered plant material (µg RUT/50 g powdered plant material) [19].

#### 2.6.5. Total Alkaloid Estimation

About 0.1 mL of sample extract was mixed in 0.4 mL HCl (2 N) and then filtered. An aliquot of 0.5 mL of this mixture was transferred into a separating funnel and rinsed 3 times with chloroform (5 mL). Then, 5 mL of 0.1% chloroform extract was initially dissolved in 5 mL of phosphate buffer with pH 7.5 and 5 mL of BTB, and then mixed with 5 mL of chloroform and shaken for 3 min. The reaction mixture was then transferred again into the separating funnel and left to sit for two hours. Then, after, the chloroform layer had collected (at the bottom of the funnel), and out of that 4 mL was taken separately and anhydrous Na_2_SO_4_ (0.2 mg) was added to that portion and shaken well to make the mixture dehydrated. After leaving for 10 min, the absorbance of the solution (supernatant) was determined at 414 nm. A standard graph of caffeine (Cf) was prepared and the TAC was represented as caffeine equivalents per 50 g of powdered plant material (µg Cf/50 g powdered plant material) [20,21].

### 2.7. Estimation of the In Vitro Antioxidant Activity

The antioxidant activity of LP stem and root extracts was estimated in vitro using DPPH, NO, H_2_O_2_ radical scavenging activities, MC, and FRAP assays.

#### 2.7.1. 2,2-Diphenyl-1-picrylhydrazyl (DPPH) Free Radical Scavenging Assay

The free radical scavenging assay of DPPH was determined utilizing the technique described by [22]. DPPH absorbs at 517 nm in its radical state, but its absorbance falls when it is reduced by another radical species or an antioxidant. To be precise, 3 mL of sample solution (stock solution of LP stem and root extracts were prepared by dissolving in methanol) at distinct concentrations (100 to 500 μg/mL) were added to 1 mL of 0.1 mM DPPH solution (weighed at 0.399 mg of DPPH and dissolved in 50 mL of methanol). The absorbance was determined at 517 nm after 30 min. Greater free radical scavenging activity indicated the lesser absorbance of the reaction solution. The 1 mL DPPH solution was added to methanol (3 mL) and the absorbance was taken as a blank. The DPPH radical scavenging activity was evaluated using the equation given below.

DPPH radical scavenging activity (%) = (Ac − As)/Ac × 100

where Ac = absorbance of ascorbic acid (control); As = absorbance in the presence of the extract.

#### 2.7.2. Nitric Oxide (NO) Scavenging Assay

The effectiveness of the NO scavenging assay was evaluated by utilizing Griess’ reagent. Briefly, 2 mL of 10 mM sodium nitroprusside in a standard phosphate buffer (pH 7.4) and 0.5 mL of sample extracts at distinct concentrations (100–500 μg/mL) were added and incubated at 25 °C for 2 h 30 min. The mixture was then kept for 5 min at 25 °C with the sulfanilic acid reagent (1 mL). The mixture was then combined with 1 mL of napthylethylene diamine dihydrochloride (0.1% *v*/*v*) and kept at RT (30 min). The absorbance was estimated at 540 nm using a UV–visible spectrophotometer [23]. The positive control was ascorbic acid.

Nitric oxide (NO) scavenging activity (%) = (Ac − As)/Ac × 100

where Ac = absorbance of ascorbic acid (control); As = absorbance in the presence of the extract.

#### 2.7.3. Hydrogen Peroxide (H_2_O_2_) Radical Scavenging Activity Analysis

H_2_O_2_ is a significant biological oxidant due to its ability to produce the hydroxyl (OH^−^) radical, i.e., a highly potent molecule. One of the most reactive oxidants in biochemical processes, the hydroxyl radical can add or remove hydrogen atoms from unsaturated hydrogen bonds present in organic lipids. However, because of its short half-life, both its efficacy and capacity to diffuse are limited. Here, 40 mM of H_2_O_2_ solution was made in phosphate buffer with pH 7.4. The prepared H_2_O_2_ solution (0.6 mL) was then added to distinct LP concentrations (10–100 µg/mL). The absorbance of the resultant solution was estimated at 230 nm. The blank was prepared with phosphate buffer only. The positive control was ascorbic acid [22]:

H_2_O_2_ radical scavenging activity (%) = (Ac − As)/Ac × 100

where Ac = absorbance of ascorbic acid (control); As = absorbance in the presence of the extract.

#### 2.7.4. Metal Chelation (MC) Activity

The colorimetric MC activity method was utilized for determining the LP extracts’ potential to chelate ferrous (Fe^2+^) ions. Briefly, plant extract (0.2 mL) with a concentration ranging from 100 to 500 µg/mL was mixed with 0.4 mL of 0.25 mM ferrozine and 0.1 mM FeSO_4_ (0.2 mL). The absorbance of the stable ferrous–ferrozine complex increased after 10 min (room temperature) at 562 nm. The positive control was EDTA [24].

Metal chelation activity (%) = (Ac − As)/Ac × 100

where Ac = absorbance of EDTA (control); As = absorbance in the presence of the extract.

#### 2.7.5. Ferric Reducing Antioxidant Power (FRAP) Assay

The FRAP assay was utilized to evaluate the antioxidant capacity of the LP extracts. The FRAP reagent was prepared with 10 mM of TPTZ in a 1:1:10 ratio with HCl (40 Mm), FeCl_3_ (20 mM), and 300 mM of acetate buffer at pH = 3.6. After incubation at room temperature for 30 min in a water bath, the different sample solution concentrations were mixed with 1 mL of the FRAP reagent, and their absorbance was determined at 593 nm. The ferric ion (Fe^3+^) was reduced to ferrous ion (Fe^2+^), which afterwards created a blue-coloured combination of Fe^2+^/TPTZ. The results were represented as µMFe(II)/g and compared with the standard (BHT) [25].

### 2.8. Elemental Analysis

For the atomic absorption spectrophotometer (AAS: Thermo Scientific, iCE 3000 Series), powdered samples were utilized. About 0.25 g of every test sample was placed in a 50 mL conical flask containing 6.5 mL of HNO_3_/H_2_SO_4_/HClO_4_ solution in a 5:1:0.5 ratio. The test sample was heated in an acidic mixture in a fume hood until the digestion was complete, as indicated by the release of white vapours from the sample flask. The distilled water (few drops) was then added, and allowed to cool at room temperature. These digested plant samples were then placed in 50 mL volumetric flasks and filled with distilled water to reach a total amount of 50 mL. The filtrate was then collected in the labelled plastic bottles after the extract had been filtered (Whatmann No. 42). The prepared mixtures were examined for the detection of desired elements by using iCE330 GF high-resolution AAS with 50 mm hollow cathode lamps. The gas pressure was air-acetylene, and different rates of gas flow were used (L/min). The proportions of various elements in the respective test samples were measured by the relevant calibration curves of standards generated by utilizing the standard solutions of Mg^2+^, Mn^2+^, Zn^2+^, and Al^3+^ [26].

### 2.9. Comparison between Conventional and Non-Conventional Methods

Conventional extraction methods are among the ancient methodologies which often involve the employment of several distinct procedures, including reflux, simple distillation, soxhlation, and cold maceration [27]. The ultrasound-assisted extraction method is a non-conventional extraction technique that can yield more product in a shorter period by using less solvent [28]. One of the most widely utilized statistical and mathematical techniques, RSM, can be utilized to optimize countless biotechnological processes and determine the impacts of multiple parameters [29,30,31,32,33,34]. In this study, ethanol is used as a sustainable solvent to optimize the extraction of alkaloids, tannins, and phenols from the stem of LP, utilizing conventional soxhlet extraction (CSE) and non-conventional ultrasound-assisted extraction (NCUSAE). In CSE, the impacts of temperature, solvent-to-substrate ratio, and extraction duration were assessed. In NCUSAE, the impact of power was also investigated. In NCUSAE, the technique can favour mass transfer procedures and save energy usage.

#### Experimental Design

The technology utilized in this research study aimed to optimize the extractive conditions of phenols, tannins, and alkaloids sequentially, utilizing ethanol as a common solvent. Two extraction methods, CSE and NCUSAE, were proposed. The impact of distinct solvent-to-substrate ratios was estimated firstly through CSE. Specifically, three factors were involved in each extraction technique, i.e., solvent-to-substrate ratios (300/10, 350/10, and 400/10 *v*/*w*); time (18, 26, and 34 h); and temperature (40, 50, and 60 °C) for CSE, and nominal power (120, 220, and 320 W); temperature (40, 50, and 60 °C); and time (30, 45, and 60 min) for NCUSAE (Supplementary Table S1). The best ratio in terms of alkaloids, tannins, and phenol extraction yields was selected to perform subsequent CSE and NCUSAE methods [35]. A central composite design (CCD) was adopted for the CSE system, taking into account temperature, time, and the solvent-to-substrate ratio as three distinct variables. Additionally, CCD was also employed for the NCUSAE. Nominal power, temperature, and time were all independent factors that were investigated. A total of 20 trials using both methods (eight cubic points + six centre point replicates + six axial points) were performed. To calculate the experimental error, the level of centre point replicates in both CCDs was utilized.

### 2.10. Analytical Methods

#### 2.10.1. Fourier-Transform Infrared Spectroscopy (FT-IR)

The extracts of LP stem and root were examined utilizing FT-IR (Bruker; Billerica, MA, USA), a specified process in the scanning wave number ranges from 4000 to 500 cm^−1^ with 4 cm^−1^ resolution in order to generate IR spectra. The data of the spectra were compared with references for determining the functional groups present in the test samples; it was possible to interpret IR spectra acquired from specific extracts [36].

#### 2.10.2. UV–Visible Spectroscopy

The characteristic peaks developed after scanning all the extracts with a UV–visible double-beam spectrophotometer 2202TS (Systronics; Ahmedabad, Gujarat, India) at wavelengths between 200 and 900 nm. Each investigation for the spectrum conformation was carried out in triplicate [37].

#### 2.10.3. GC-MS

In the analysis, the contents were separated using gas chromatography (Thermo Scientific, TSQ 8000 Evo; Waltham, MA, USA) with a fused silica column comprising Elite-5 Mass spectroscopy. Helium (He) was used as a carrier gas with a constant flow rate (1 mL/min). The injector was adjusted at a temperature of 260 °C for the duration of the chromatography run. The extracted sample (1 μL) was placed in the instrument, and the oven temperature was adjusted to 60 °C for 2 min, 300 °C/min at a rate of 10 °C, and 300 °C for 6 min. The specific conditions of the mass detector were as follows: the ionization mode electron impact (70 eV), the scanning period (0.2 s), and the scanning interval (0.1 s) for fragments between 40 and 600 Dalton. The temperature of both the transfer line as well as ion source was 240 °C. The database of known spectral components from the GCMS NIST (2008) library was utilized for comparing the spectral components [38].

### 2.11. Statistical Calculations

All statistical calculations were made in triplicate, and the outcomes were calculated as mean values with standard error (mean ± SE). Phytoconstituent levels and antioxidant activity were compared on average, and these data were then analysed using analysis of variance (ANOVA) in Graph Pad Prism 8 and IBM SPSS Statistics 20 software. Figures of quantitative analyses and in vitro antioxidant assays were made by using Graph Pad Prism 8 software. A value of * *p* < 0.01 indicated significance, ** *p* ˂ 0.001 high significance, and *** *p* ˂ 0.0001 very high statistical significance. Tukey’s one-way ANOVA test was utilized to determine the differences among groups. Minitab 18.1 software was employed to carry out the ANOVA, RSREG (Response Surface Regression), and respective significance tests. ANOVA was used to figure out how accurately and efficiently the experimental findings fit the created polynomial model. The analysis of UV–visible experimental results was performed by utilizing Origin Pro 8.5 software.

## 3. Results and Discussion

### 3.1. Extraction Yield Values of LP Extracts

The percentage yields (% *w*/*w*) of the LP stem and root extracts were analysed using different solvents. This indicated the existence of various biologically active components within all extracts of the experimental plant. The findings revealed that in cases of both stem and root, the polar solvent ethanol had a high extractive yield (7.37 ± 0.11 and 5.70 ± 0.02). The pH of LP stem and root extracts (1 mg/mL) was weakly acidic (pH~5–6), i.e., 5.75 ± 0.27 (stem) and 5.11 ± 0.13 (root). Suitable climatic conditions for the collection of LP are from August to January, when plants bloom and produce fruit. The plants may thrive in dry places with exceptionally harsh climates, ranging from −0.4 to 49.5 °C, and sparse, irregular, and variable rainfall [39].

### 3.2. Physicochemical Analysis of LP

In the present study, the total ash value (TAV) was noted to be higher in the stem (9.96 ± 1.02) than in the root (7.51 ± 0.31). Higher water-soluble ash (WSA) was observed in the stem (3.53 ± 0.45), as compared to the root (3.25 ± 0.56). Higher acid-insoluble ash (AIA) was also observed in the stem (3.53 ± 0.45), as compared to the root (3.59 ± 0.34). Foreign organic matter was observed to be higher in the stem (0.24 ± 0.05) as compared to the root (0.53 ± 0.03). By using the loss on drying (LOD) method at 105 °C, the moisture content was discovered to be higher in the stem (28.36 ± 1.88) than in the root (8.93 ± 1.14). The amount of crude fibre was estimated to be higher in the stem (5.29 ± 0.41) than in the root (1.54 ± 0.44). The amount of crude fat was estimated to be higher in the stem (4.58 ± 0.63) than in the root (3.11 ± 0.18). In Qatar, a study was conducted to discover the proximate mass composition of the aerial part of LP, showing 6.77% moisture, 4.69% ash, 5.60% protein, 15.61% carbs, and 4.39% crude fibre [40].

### 3.3. Qualitative Screening of Phytochemical Compounds in Various LP Extracts

The plant contains secondary metabolites like alkaloids, flavonoids, terpenoids, glycosides, etc., in an appropriate amount as compared to primary metabolites like proteins, amino acids, carbohydrates, and lipids, as shown by the results of preliminary qualitative screening of distinct phytocompounds within the LP extracts. These metabolites were found in petroleum ether, hexane, chloroform, ethyl acetate, and ethanol solvents, indicating that these solvents can be used to isolate the particular bioactive chemicals effectively from LP extracts (Supplementary Table S2). The results showed that LPSE has a good amount of primary as well as secondary metabolites compared to other LP extracts.

In all, there were approximately 273 phytocompounds (50 phytocompounds were extracted) identified through a preliminary phytochemical screening in distinct parts of LP that involved fatty acids, peptides, sterols, tannins, terpenes, fatty acid esters, phenolics, cardiac glycosides, alkaloids, flavonoids, pregnane glycosides, sugars, amino acids, simple amines, and hydrocarbons. Out of the total recorded LP phytocompounds, 41, or the majority of them, were hydrocarbons (alkanes, alkenes, aromatic hydrocarbons, alcohols, and ketones) [41,42,43,44,45]. Studies have been carried out on the callus of LP that showed the highest concentration of soluble carbohydrates. In contrast, the stems and leaves of LP had the highest concentrations of starch, protein, and phenolic compounds, respectively [46]. A further investigation carried out in the UAE and Egypt found that the 95% ethanol extract of the aerial part of LP included anthraquinone, terpenes, sterols, saponins, tannins, and flavonoids [47,48].

### 3.4. Quantitative Evaluation of Phytochemical Compounds in Various LP Extracts

#### 3.4.1. Evaluation of Total Phenolic (TPC) Content

**Figure 1:**
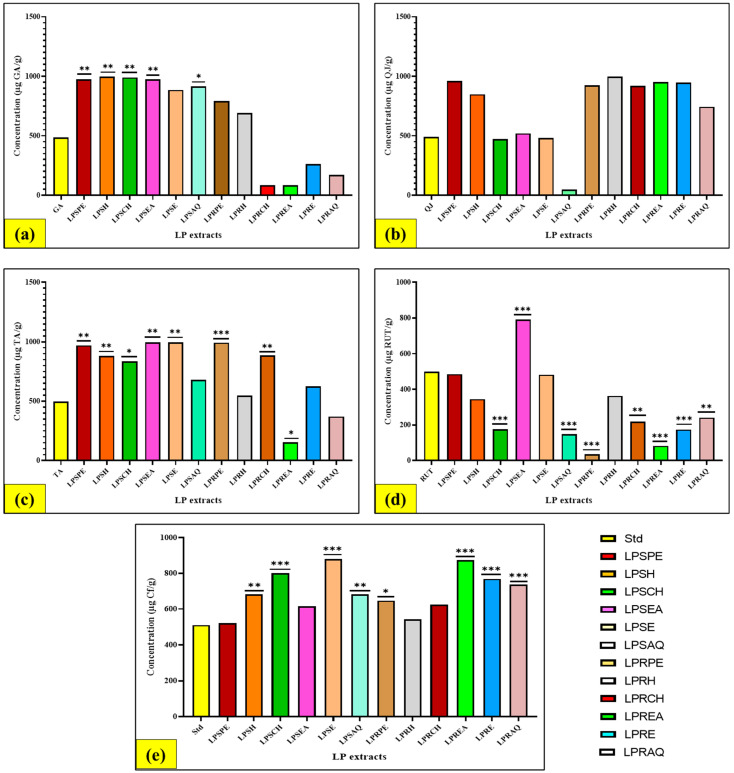
Comparative graph of the quantitative analysis between two parts of LP (stem and root): (**a**) TPC, (**b**) TSC, (**c**) TTC, (**d**) TFC, (**e**) TAC. (LPSPE, *L. pyrotechnica* stem petroleum ether; LPSH, *L. pyrotechnica* stem hexane; LPSCH, *L. pyrotechnica* stem chloroform; LPSEA, *L. pyrotechnica* stem ethyl acetate; LPSE, *L. pyrotechnica* stem ethanolic; LPSAQ, *L. pyrotechnica* stem aqueous; LPRPE, *L. pyrotechnica* root petroleum-ether; LPRH, *L. pyrotechnica* root hexane; LPRCH, *L. pyrotechnica* root chloroform; LPREA, *L. pyrotechnica* root ethyl-acetate; LPRE, *L. pyrotechnica* root ethanolic; LPRAQ, *L. pyrotechnica* root aqueous.) Note: values with * (0.01); ** (0.001); *** (0.0001) superscripts were significant (*p* < 0.05).

The TPC was estimated in all stem and root extracts by using the standard curve’s regression equation (y = 0.0014x + 0.0086, R^2^ = 0.9946). The TPC was estimated in all extracts of stem and root by using the regression equation of the standard curve (y = 0.0014x + 0.0086, R^2^ = 0.9946). There was a remarkable variation in TPC present in both stem and root parts of LP, ranging from 83.62 to 996.24 µg GA/50 g dried extract. Different fractions of LP (500 µg/mL) contained phenol content in the respective sequence LPSE (*L. pyrotechnica* stem ethanolic) > LPSH (*L. pyrotechnica* stem hexane) > LPSPE (*L. pyrotechnica* stem petroleum ether) > LPSEA (*L. pyrotechnica* stem ethyl acetate) > LPSAQ (*L. pyrotechnica* stem aqueous) > LPSCH (*L. pyrotechnica* stem chloroform) > LPRPE (*L. pyrotechnica* root pet-ether) > LPRH (*L. pyrotechnica* root hexane) > GA (gallic acid) > LPRE (*L. pyrotechnica* root ethanolic) > LPRAQ (*L. pyrotechnica* root aqueous) > LPREA (*L. pyrotechnica* root ethyl-acetate) > LPRCH (*L. pyrotechnica* root chloroform). The maximum level of phenols was shown by the LPSE extract (996.24 ± 66.81 µg GA/50 g dried extract) in LP stem, and the highest phenol level was shown by the LPRPE extract (793.14 ± 2.12 µg GA/50 g dried extract) in the LP root (Figure 1a).

In one study, it was reported that LP shoots have more lipids, total soluble phenols, and sugar contents (140, 5.5, and 0.26 mg/g dry weight), whereas the roots, on the other hand, contained 15 and 4 mg of protein and starch per g dry weight, respectively [49]. In another study, the TPC of the methanol extract of LP, together with its hexane, ethyl acetate, and aqueous fractions, was investigated using the Folin–Ciocalteu assay. The hexane fraction exhibited the largest level, measuring 25.79 ± 0.11 mg GA/g dry material, whereas the ethanol extract of the aerial parts of LP showed the highest phenol level (158.3 ± 6.25 mg GA/extract) in Egypt [50]. In India, Purohit [45] found that the ethanol extract of the whole LP plant contained 49.47 mg GA/g DW of phenols. Furthermore, the total polyphenolic content of the ethanol crude extract, ethyl acetate, n-butanol, and aqueous fractions of the aerial parts of LP were investigated using previously stated methods. The results revealed that the ethyl acetate fraction displayed a higher phenolic content (252.27 ± 2.84 mg GA/g of dry extract) than other fractions [51]. Moreover, the TPC of the hexane fraction of the aerial parts of LP was studied in other research, and it was measured to be 10.53 mg GA/g of dry extract [52]. An additional study undertaken in Pakistan showed that the TPC was found to be 2.11 ± 0.86 mg GA/g in the 80% methanol extract of the LP leaves [53]. Interestingly, the 70% ethanol extract of the LP green fruits was examined in Egypt for TPC, and it was found to be 59.1 mg GA/g of dry material [54].

#### 3.4.2. Evaluation of Total Saponin (TSC) Content

The TSC was estimated in stem and root extracts by using the standard curve’s regression equation (y = 0.0007x + 0.1103, R^2^ = 0.9935). The TSC of LP was calculated by analysing the purple colour produced by the reaction that occurs between saponin and acid. In both plant parts, the total saponin content was generally determined to be moderately high (*p* < 0.05). The LPSPE extract of the stem showed the greatest quantity of saponin content (783.38 + 127.84 g QJ/50 g dried extract), while the LPREA extract of the root (846.24 ± 119.33 µg QJ/50 g dried extract) showed the maximum saponin content (Figure 1b). The sequence of TSC in distinct LP fractions was as follows: LPREA > LPRPE > LPSPE > LPRH > LPRAQ > LPRE > LPSH > LPRCH > LPSEA > QJ > LPSE > LPSAQ > LPSCH. It was evident after the quantitative screening of different parts of LP that the aerial part showed higher saponin content, representing 0.46% compared to other phytocompounds [55].

#### 3.4.3. Evaluation of Total Tannin (TTC) Content

The TTC was estimated in all stem and root extracts by using the standard curve’s regression equation (y = 0.0003x + 0.0552, R^2^ = 0.9977). The findings of the current quantitative assay showed a broad range of remarkable variations in the TTC within the distinct parts of LP, ranging from 153.78 to 997.11 µg TA/50 g dried extract. The sequence of TTC in distinct LP fractions was as follows: LPSE > LPSEA > LPRPE > LPSPE > LPRCH > LPSH > LPSCH > LPSAQ > LPRE > LPRH > LPRAQ > TA > LPREA.

The LPSE extract of the stem contained maximum tannins (*p* < 0.05) compared to the other LP extracts. The LPSE extract in the case of the stem (997.11 ± 31.76 µg TA/50 g dried extract) and the LPRPE extract in the case of the root (993.77 ± 68.75 µg TA/50 g dried extract) showed a maximum level of tannin when compared to the other solvents (Figure 1c). It was demonstrated after the quantitative screening of distinct parts of LP that the aerial part showed higher tannin content, representing 154.961 mg of TAE/100 g of extract compared to other phytocompounds [55].

#### 3.4.4. Evaluation of Total Flavonoid (TFC) Content

The TFC was estimated in all stem and root extracts by using the standard curve’s regression equation (y = 0.0025x + 0.0191, R^2^ = 0.9979). The outcomes represented a remarkable difference in the TFC within the different LP extracts, ranging from 148.23 to 791.43 mg QJ/50 g dried extract. The sequence of TFC in distinct LP fractions was as follows: LPSEA > RUT > LPSPE > LPSE > LPRH > LPSH > LPRAQ > LPRCH > LPRE > LPSAQ > LPSAQ > LPREA > LPRPE. Among the studied LP parts, the maximum flavonoid content was found in the LPSEA extract of the stem with a value of 791.43 ± 3.53 µg RUT/50 g dried extract, followed by the LPRH extract of the root (363.16 ± 3.78 µg RUT/50 g dried extract) (Figure 1d). In one study, the TFC of the methanol extract, together with its hexane, ethyl acetate, and aqueous fractions, was investigated using the AlCl_3_ assay. The hexane fraction exhibited the largest level, measuring 20.64 ± 0.33 mg RE/g [50]. In contrast, the ethanol extract of the aerial parts of LP showed the highest level of flavonoids (89.0 ± 3.40 mg QE/g extract) in Egypt [56]. In India, Purohit [45] found higher TFC content in the ethanol extract of the whole LP plant, with a value of 34.85 mg QE/g dried extract.

#### 3.4.5. Evaluation of Total Alkaloid (TAC) Content

The TAC was estimated in all stem and root extracts by using the standard curve’s regression equation (y = 0.0035x + 0.0173, R^2^ = 0.9913). The outcomes of the current assay showed a broad range of remarkable differences in TAC within the distinct parts of LP, ranging from 520.10 to 966.48 µg TA/50 g dried extract. The sequence of TAC in distinct LP fractions was as follows: LPSE > LPREA > LPSCH > LPRE > LPRAQ > LPSAQ > LPSH > LPRPE > LPRCH > LPSEA > LPRHA > LPSPE > Cf. The LPSE extract of the stem (966.48 ± 24.51 µg TA/50 g dried extract) and the LPREA extract of the root (874.96 ± 5.92 µg TA/50 g dried extract) showed a maximum level of alkaloid compared to the other solvent systems (Figure 1e). In a study, the results of quantitative screening of all parts of LP showed that the roots exhibited a greater amount of alkaloids (3.267%) than other parts of LP [57].

### 3.5. Assessment of In Vitro Antioxidant Activities of Different LP Extracts

Recent studies on the antioxidant properties of the LP (whole plant), methanol extract and its ethyl acetate, hexane, and aqueous fractions have been utilized for some antioxidant assays, namely, ABTS, DPPH, CUPRAC, FRAP, MC, and total antioxidant (phosphomolybdenum assay). In the DPPH and ABTS scavenging assays, ethyl acetate had the greatest capacity to scavenge free radicals compared to other solvents. Studies have been undertaken on the non-phenolic phytocompounds of LP, showing that these phytocompounds are the most effective chelating agents for metal ions. In addition, multivariate research also suggested that the strong antioxidant capacity of LP was a result of its phenolic and terpenoid composition [50,58].

#### 3.5.1. DPPH Free Radical Scavenging Assay

**Figure 2:**
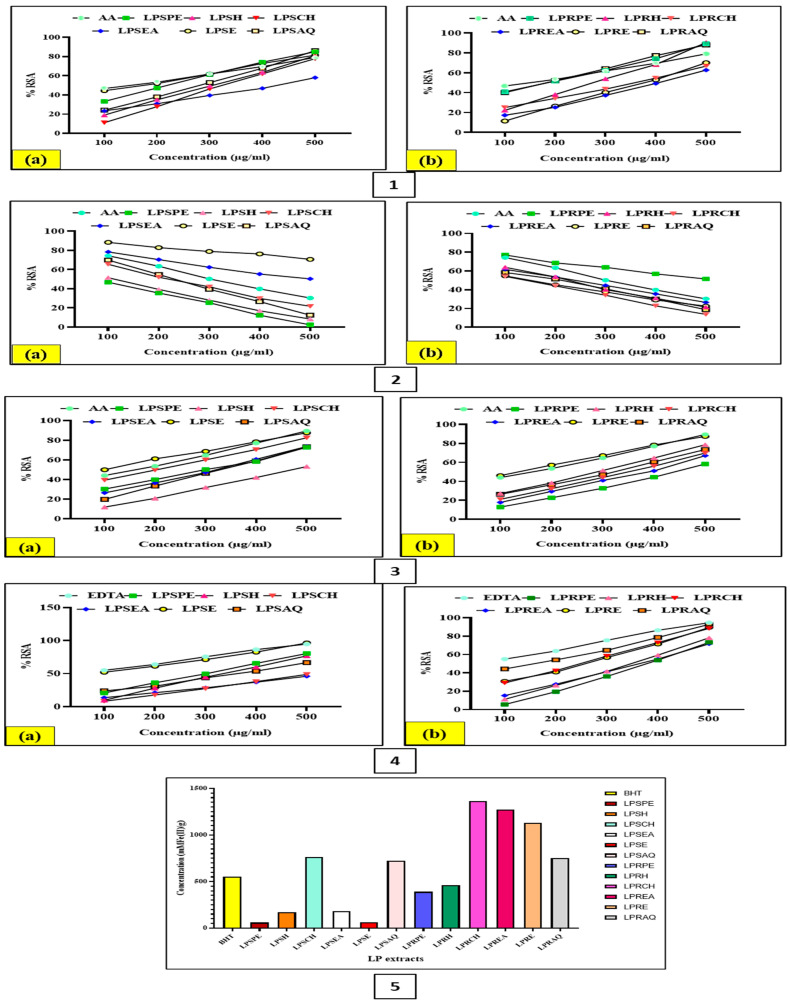
In vitro antioxidant assays of both parts of LP: (**1**) DPPH scavenging assay, (**2**) NO scavenging assay, (**3**) H_2_O_2_ scavenging assay, (**4**) MC assay, (**5**) FRAP assay. Note: (**a**) LPSPE, LPSSH, LPSCH, LPSEA, LPSE, and LPSAQ extracts of LP stem; (**b**) LPRPE, LPRH, LPRCH, LPREA, LPRE, and LPRAQ extracts of LP root.

The antioxidant potential of LP was evaluated by utilizing the DPPH free radical scavenging assay. The absorbance data were recorded against the chosen concentrations (100, 200, 300, 400, 500 µg/mL). The antioxidant potential of the standard and extract is shown by the IC_50_ values for the standard ascorbic acid (AA) and all of the LP extracts. In this work, the DPPH free radical scavenging activity of LP and the standard AA with 500 µg/mL concentration were observed to be in the following sequence: LPSE > LPRAQ > LPRPE > LPSPE > LPRH > LPSAQ > LPSH > LPSCH > LPRCH > LPRE > LPREA > LPSEA. The highest IC_50_ values in the case of stem and root extracts were 43.6 ± 95.71 µg/mL and 368.8 ± 47.61 µg/mL, respectively, indicating the lower antioxidant capability of the samples, whereas the lowest IC_50_ value of the LPSE extract of the stem, i.e., 170.4 ± 19.67 µg/mL, showed maximum antioxidant potency as compared to the standard (AA) (Figure 2 (1)). The LP potency was demonstrated by the results of the DPPH scavenging activity experiment in this investigation. Based on this, the LPSE extracts might have contained substances that may help a free radical to get rid of its odd electron, which makes it reactive.

A study was conducted to determine the antioxidant capacity of the ethanol extract of whole LP plant and its fractions, including petroleum ether, chloroform, ethyl acetate, and methanol fractions, which were evaluated by performing a DPPH free radical scavenging assay in which quercetin and GA were utilized as standards. The ethanol extract and its fractions showed the best antioxidant activity at 100 µg/mL concentration [45]. The methanol extract of the whole LP plant was also investigated by utilizing a DPPH assay for its free radical scavenging activity in vitro. The finding demonstrated that the methanol extract significantly reduced the percentage of free radicals in a dose-dependent manner, i.e., the IC_50_ value for the DPPH free radical scavenging assay was found to be 152.46 µg/mL due to the activity of the polyphenolic content and other phytocompounds which exist in LP [22].

By employing the DPPH assay, the antioxidant effect of an 80% aqueous-methanol extract of the LP leaves was examined. The IC_50_ value for the DPPH assay was found to be 991.62 μg/mL [53]. It is interesting to note that, when tested in several assays for its antioxidative impact, the 70% aqueous-ethanol extract of Egyptian green LP fruits showed a good dose-dependent scavenging activity with an IC_50_ value of 89.3 μg/mL in the DPPH assay [54]. Furthermore, methanol extracts of the roots and aerial parts of LP demonstrated a notable capacity to scavenge free radicals, which increased with concentration when compared to BHA, a common synthetic antioxidant. The investigation assessed this activity using an in vitro test, namely the DPPH assay, which showed that the crude extract of the LP root exhibited stronger activity than that of the aerial portions up to a concentration of 40 µg/mL [57]. The antioxidant effect of the ethanol extract of the Egyptian LP aerial parts was evaluated through DPPH assay using Trolox as a reference, and the results were expressed as Trolox equivalent antioxidant capacity (TEAC). The extract displayed high antioxidant and DPPH radical scavenging activities (1.84 TEAC) [56].

#### 3.5.2. Hydrogen Peroxide (H_2_O_2_) Radical Scavenging Activity Analysis

A few enzymes can be immediately inactivated by H_2_O_2_, a weak oxidizing agent, typically by the oxidation of crucial thiol (-SH) groups. It can quickly pass across cell membranes. Many of the adverse consequences of H_2_O_2_ may originate from its ability to react with Fe^2+^ and/or Cu^2+^ ions once it is within the cell, forming hydroxyl radicals. Therefore, it is advantageous for cells biologically to regulate the amount of H_2_O_2_ that accumulates. The ability of the plant extract to scavenge H_2_O_2_ may be due to its phenolics, which give H_2_O_2_ an electron and convert it to water. H_2_O_2_ might be scavenged by the extract in a concentration-dependent way [59].

The inhibition data were obtained in relation to the chosen concentrations (100–500 µg/mL). Plots of the standard inhibition curve for the ability of ascorbic acid to scavenge H_2_O_2_ radical and for all the plant extracts of LP were constructed. In this work, the H_2_O_2_ radical scavenging activity of LP and the standard (AA) with 500 µg/mL concentration was observed to be in the following sequence: LPSE > LPRE > LPRCH > LPRH > LPSPE > LPSEA > LPSAQ > LPRCH > LPREA > LPRAQ > LPRPE > LPSH. Using a regression equation, the IC_50_ values of ascorbic acid and the percentage (%) inhibition of H_2_O_2_ radical scavenging of plant fractions were determined. The highest IC_50_ values in the case of stem and root extracts were 470.7 ± 56.02 µg/mL and 447.9 ± 43.24 µg/mL, respectively, indicating the lower antioxidant capability of the samples (Figure 2 (3)), whereas the lowest IC_50_ value of the LPSE extract of stem, i.e., 89.87 + 50.41 µg/mL showed the highest antioxidant activity as compared to the standard (AA).

In a study, the methanol extract of the whole LP plant was investigated by utilizing the H_2_O_2_ assay for its free radical scavenging activity in vitro. The finding demonstrated that the methanol extract significantly reduced the percentage of free radicals in a dose-dependent manner, i.e., the IC_50_ value for the H_2_O_2_ free radical scavenging assay was found to be 65.48 µg/mL due to the activity of the polyphenolic content and other phytocompounds that exist in LP [22]. Moreover, when compared to BHA, a well-known synthetic antioxidant, methanol extracts from the roots and aerial sections of LP showed a noteworthy potential to scavenge free radicals that enhanced with concentration. Using an in vitro test called the H_2_O_2_ assay, the investigation evaluated this activity and found that, up to a concentration of 40 µg/mL, the crude extract of the LP root exhibited greater activity than that of the aerial sections [57].

#### 3.5.3. Metal Chelation Activity

Here, we checked the chelating capacity of iron in both the stem and root parts of LP. All fractions represented a capacity to chelate the metal ions. The IC_50_ values of the LP extracts and EDTA (standard) for chelating iron metal with 500 µg/mL concentration were observed to be in the following sequence: LPSE > LPRAQ > LPRE > LPRCH > LPSPE > LPSH > LPRH > LPRPE > LPSAQ > LPREA > LPSEA > LPSH. The findings of the chelating metal iron showed that the LPSE extract of the stem (86.06 ± 42.67 µg/mL) had the highest and appropriate capacity (*p* < 0.05) to chelate the metal ion, while the LPSCH extract of the stem exhibited the lowest ability, with an IC_50_ value of 657.1 ± 183.4 µg/mL (Figure 2 (4)). In a recent study, MC activity was examined to study the antioxidant capacity of the methanol extract of the whole LP plant and its ethyl acetate, H, and aqueous fractions by using EDTA as a standard. The sharpest MC effect was found in the hexane fraction (11.57 ± 0.29 mg EDTA/g dry extract) [58].

#### 3.5.4. Nitric Oxide (NO) Scavenging Assay

The NO scavenging assay of the LP test fractions and the standard (AA) with 500 µg/mL concentration was observed to be in the following sequence: LPSE > LPRPE > LPSEA > LPREA > LPRE > LPSCH > LPRH > LPRAQ > LPRCH > LPSAQ > LPSH > LPSPE. The LPSE extract of the stem exhibited the lowest IC_50_ value (70.9 ± 9.91 µg/mL), followed by other test fractions, indicating strong NO scavenging activity with the lowest concentration to obtain 50% radical scavenging power. In the case of the stem, the IC_50_ values of LPSPE, LPSH, LPSCH, and LPSAQ extracts were lower than those of the standard (AA) (Figure 2 (2)). In the case of the root, the IC_50_ values of LPRH, LPRCH, LPREA, LPRE, and LPRAQ extracts were lower when compared to the standard (AA). Therefore, it was indicated that these fractions have greater scavenging power when compared to the AA.

Two categories can be used to classify plant tannins. According to Yoshiki et al. [60], hydrolysed tannins contain remnants of polyol and ellagic tannins that are not entirely degraded. According to Bernard et al. [61], plant tannins are found in a wide range of plant species, particularly in grains, shrubs, herbages, and medicinal resources. Many applications in food production and medical areas make use of tannins’ antioxidant qualities. To determine the pertinent antioxidant activity of tannins, numerous investigations have been carried out in recent years. Tannins have garnered a lot of interest because of their antioxidant properties, which can prevent cancer, heart disease, and osteoporosis. Excellent antibacterial and antioxidant properties of tannins can help to alleviate the symptoms of urinary tract infections. According to reports, the amount of hydroxyl groups in tannins and the hydrogen peroxide production are significant markers for assessing the antibacterial qualities, which are positively associated with antioxidant qualities. Certain studies have suggested that tannins can shield against viruses like norovirus, human immunodeficiency virus (HIV), and bovine adeno-associated virus (BAAV) [62]. A study conducted by Masood et al. [63] determined that the TTC of LP was 0.54 ± 0.01 mg TAE/g dry weight of the extract.

#### 3.5.5. Ferric Reducing Antioxidant Power (FRAP) Assay

The FRAP assay was estimated by the ferric reducing capacity of the standard (BHT) by using the regression equation of the standard curve (y = −0.0849x + 97.32, R^2^ = 0.9915) and the plant extracts. In this study, the reducing capacity of LP and the standard (BHT) with 500 µg/mL concentration was observed to be in the following sequence: LPRCH > LPREA > LPRE > LPSCH > LPSAQ > LPRAQ > LPRH > LPSH > LPSE > LPRPE > LPSEA > LPSE. The LPSCH extract of the stem (59.36 ± 0.02 μMFe(II)/g) and the root (69.75 ± 0.01 μMFe(II)/g) revealed enhanced antioxidant potential compared to the other extract fractions. The FRAP activity of the LP extracts was lower compared to the BHT, but was significant (*p* < 0.05). As shown in Figure 2 (5), the LPSEA extract of the stem and the LPRE extract of the root showed lower ferric ion-reducing power ability.

Using EDTA as a reference, a FRAP assay was recently performed in a study to investigate the antioxidant potential of the methanol extract of the entire LP plant, as well as its ethyl acetate, hexane, and aqueous fractions. The extract was used at dosages of 0.5–5 mg/mL. In the FRAP test, it exhibited the maximum activity (117.42 ± 1.28 mg TE/g extract) [58]. Employing the FRAP assay, the antioxidant capacity of the 80% aqueous-methanol extract of the LP leaves was evaluated. The results revealed IC_50_ 1.69 mMol Fe^+2^/g for the FRAP assay [53]. Furthermore, using the FRAP method, the antioxidant activity of the hexane fraction of the aerial parts was examined. The results were compared to those of the ethanol extract and its ethyl acetate, butanol, and aqueous fractions, which had previously been documented. According to the FRAP assay results, the hexane fraction had the lowest antioxidant capacity (0.049 µmol/mg) [52]. Moreover, the FRAP assay was also used to determine the antioxidant impact of the ethanol extract of LP aerial parts, as well as its ethyl acetate, butanol, and aqueous fractions. The IC_50_ value of the ethyl acetate fraction displayed the best antioxidant activity (1.2 mmol ascorbic acid equivalent/g in the FRAP) [51].

### 3.6. Correlation of Phytochemical Contents with Their In Vitro Antioxidant Activities

**Figure 3:**
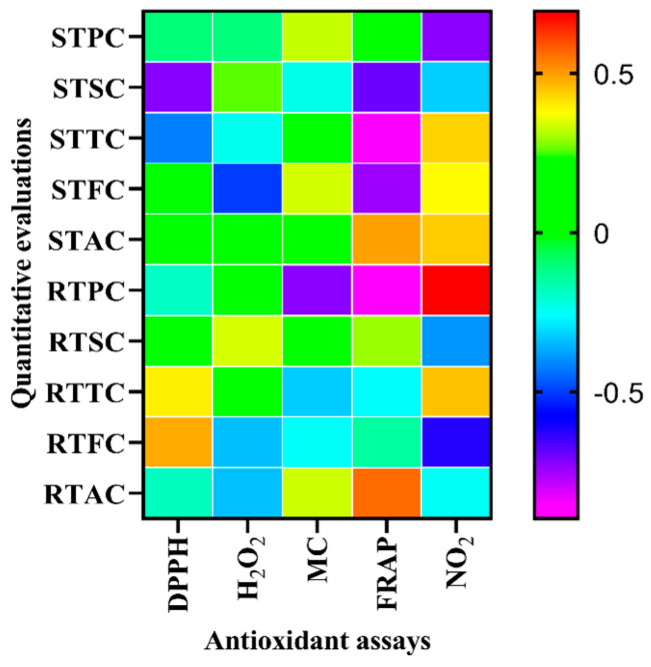
Heat map showing the correlation of phytochemicals with their in vitro antioxidant activities. Note: for stem (STPC, STSC, STTC, STFC, STAC) and root (RTPC, RTSC, RTTC, RTFC, RTAC).

The coefficients of determination (R^2^) between antioxidant potential and phytochemical components were evaluated in both the stem and root parts of LP (Figure 3). In the case of the stem, the outcomes showed a strong and significant correlation between TTC and FRAP (R^2^ = −0.849*) (*p* < 0.05), but a weak one for TTC and DPPH (R^2^ = −0.423), TTC and H_2_O_2_ (R^2^ = −0.241), TFC and MC (R^2^ = 0.158), and TTC and NO (R^2^ = 0.431). Phenol content showed a strong correlation with NO, a moderate correlation with metal chelation, and a weak correlation with DPPH, H_2_O_2,_ and FRAP; the saponin content exhibited a strong correlation with FRAP and DPPH, while a moderate correlation with H_2_O_2_, MC, and NO; and the alkaloid content exhibited a strong correlation with NO and FRAP, but a moderate correlation with MC and a weak correlation with DPPH and H_2_O_2_ in the case of the LP stem. A strong and significant correlation was found between TFC and FRAP for the stem (R^2^ = −0.746*) (*p* < 0.05), while a moderate one was found for TTC and NO (R^2^ = 0.336) and TTC and NO (R^2^ = 0.38), and a weak one for TFC and DPPH (R^2^ = 0.079) (*p* < 0.05) and TFC and H_2_O_2_ (R^2^ = −0.503).

In the case of the root, there was a significant and strong correlation between the antioxidant activity determined by TPC with FRAP (R^2^ = −0.898*) (*p* < 0.05), MC (R^2^ = −0.729) and NO (R^2^ = 0.694), while a weak one with DPPH (R^2^ = −0.192) and H_2_O_2_ (R^2^ = 0.128). A moderate as well as non-significant correlation resulted between TSC with H_2_O_2_ (R^2^ = 0.339) and NO (R^2^ = −0.394), while a weak correlation with DPPH (R^2^ = 0.111), MC (R^2^ = 0.220) and FRAP (R^2^ = 0.294) was found. A strong correlation was shown by TTC with DPPH (R^2^ = 0.396), MC (R^2^ = −0.327) and NO (R^2^ = 0.453), while a moderate one was shown with FRAP (R^2^ = −0.259), and a weak one with H_2_O_2_ (R^2^ = 0.073). In the case of TFC, a strong correlation was observed with DPPH (R^2^ = 0.482), H_2_O_2_ (R^2^ = −0.343) and NO (R^2^ = −0.623), a moderate correlation with MC (R^2^ = −0.255), and a weak correlation with FRAP (R^2^ = −0.147). A strong correlation was found between TAC and FRAP (R^2^ = 0.560), TAC and H_2_O_2_ (R^2^ = −0.341), and TAC and MC (R^2^ = 0.330), a moderate correlation was observed for TAC and NO (R^2^ = −0.250), and a weak correlation was shown for TAC and DPPH (R^2^ = −0.184).

### 3.7. Elemental Analysis

The elemental analysis was carried out by using the comparator method of the AAS technique in mg/L dry weight of the plant materials. The findings indicated that the LP root section has the highest content of Mg (4.0665 mg/L), compared to Al (0.999 mg/L), Mn (0.3694 mg/L), and Zn (0.1295 mg/L), whereas the stem has the highest concentration of Al (4.7917 mg/L) compared to Mg (4.3314 mg/L), Mn (0.7600 mg/L), and Zn (0.2649 mg/L). A study reported the mineral content analysis, showing the mineral content in the fruits; i.e., the fruits contained 156 mg of calcium, 317 mg of phosphorus, and 3.18 mg of iron and the pulp had 68 calories per 100 g [64].

### 3.8. Extraction Using Conventional Soxhlet Extraction (CSE) and Non-Conventional Ultrasound-Assisted Extraction (NCUSAE) Methods

#### 3.8.1. Extraction Using the Conventional Soxhlet Extraction (CSE) Method

Based on the outcomes of the quantitative estimation of secondary plant metabolites in the stem and root of LP, it was concluded that the total TPC, TTC, and TAC contents of LPSE extract gave better results than the other quantitative assays. Due to this, the optimization of the extraction methodology of these metabolites could be possible by comparing CSE with NCUSAE using LPSE extract. In general, distinct factors like solvent-to-substrate ratio, extraction time, and temperature are involved in the CSE of materials to facilitate the breakdown of cellular tissues and promote mobility (Table 1). The highest amounts of TPC and TTC content were extracted at a 300/10 solvent/material (S/M) ratio, whereas the TAC was extracted best at a 400/10 S/M ratio. From this, it can be concluded that differences in the solvent-to-substrate ratio had a remarkable impact on the extractive yields of bioactive compounds from the LP stem.

**Figure 4:**
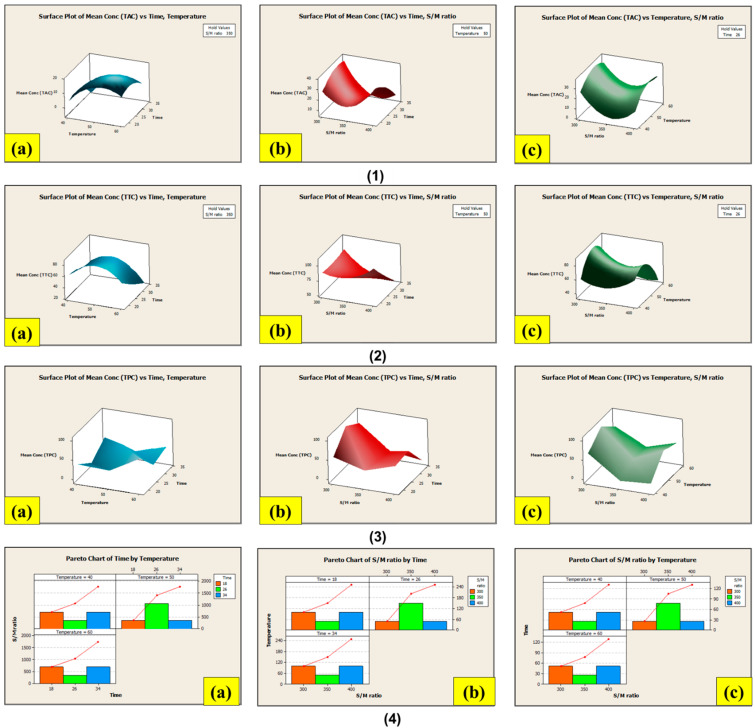
Response surface contour of the central composite design for CSE of (**1**) TAC, (**2**) TTC, (**3**) TPC; (**4**) pareto chart of central composite design for the CSE of extraction yield. Note: (**a**) time (h) vs. temperature (°C) (**b**) time (h) vs. S/M ratio (mL/g), (**c**) temperature (°C) vs. S/M ratio (mL/g).

Consequently, in association with the findings observed both for enhancing the extractive yields as well as solvent usage, the solvent-to-substrate ratio selected for the CSE method was a 300/10 S/M ratio for TPC and TTC and a 400/10 S/M ratio for TAC. The maximum experimental value achieved for TPC and TTC was found to be 102.71 ± 0.009 µg GA/50 g and 95 ± 0.005 µg TA/50 g, after 26 h at 50 °C. The highest experimental value achieved for TAC was found to be 38.48 ± 0.004 µg Cf/50 g, after 18 h at 60 °C. The highest experimental value obtained for total yield was found to be 21.92%, after 18 h at 40 °C with a 400/10 S/M ratio. As a result, CSE focused on estimating the impact of the differences in time and temperature on the extraction of the metabolites discussed earlier. The model equations for the extraction of these chemicals (Equations (1)–(3)) are provided below, and the associated modelled response surface is shown in Figure 4:

TPC (mg GA/g) = 1392.67 + (−12.60) × S/M + 19.15 × T + 32.42 × t + 0.02 × S/M^2^ + (−0.21 × T)^2^ + (−0.21 × t)^2^ + 0.02 × S/M × T + (−0.05) × S/M × t + (−0.12) × T × t (1)

TTC (mg TA/g) = −586.179 + (−1.565) × S/M + 32.172 × T + 13.813 × t + 0.005 × S/M^2^ + (−0.236 × T)^2^ + (−0.033 × t)^2^ + (−0.020) × S/M × T + (−0.038) × S/M × t + (−0.080) × T × t (2)

TAC (mg Cf/g) = 413.469 + (−4.027) × S/M + 7.149 × T + 10.639 × t + 0.006 × S/M^2^ + (−0.079 × T)^2^ + (−0.078 × t)^2^ + 0.003 × S/M × T + (−0.022) × S/M × t + 0.009 × T × t (3)

where S/M is solvent to material ratio, t is time (h), and T is temperature (°C).

#### 3.8.2. Extraction Using the Non-Conventional Ultrasound-Assisted Extraction (NCUSAE) Method

**Figure 5:**
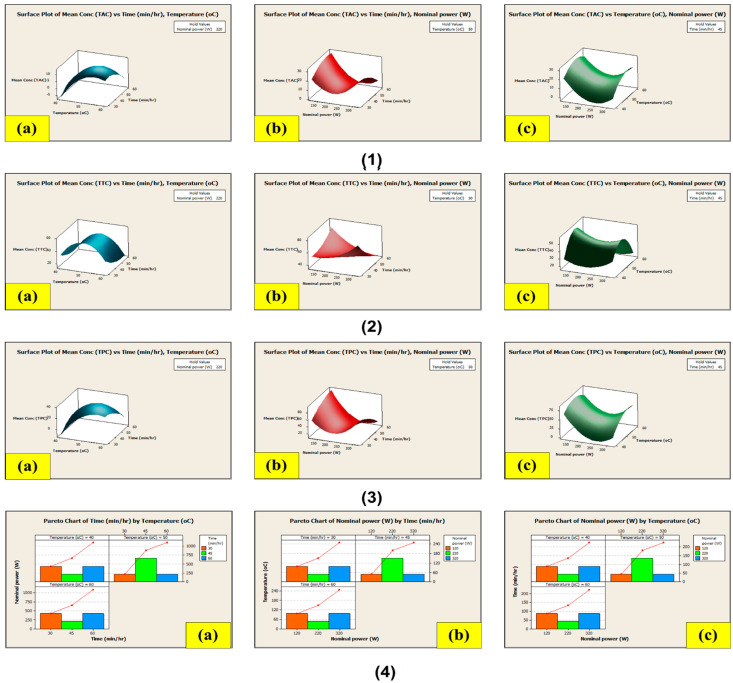
Response surface contour of the central composite design for NCUSAE of (**1**) TAC, (**2**) TTC, (**3**) TPC; (**4**) pareto chart of central composite design for NCUSAE of extraction yield. Note: (**a**) time (h) vs. temperature (°C); (**b**) time (h) vs. nominal power (W); (**c**) temperature (°C) vs. nominal power (W).

The second extraction method relied on solid–liquid extraction incorporating ultrasounds, combining the effects of time, temperature, power, and frequency to create a cavitation phenomenon in the biomass and facilitate the extraction of biologically active substances. This method utilized the same S/M ratio (300/10) as all three previously mentioned factors. In this case, the highest extraction values for TPC and TAC from the LP stem were 90.98 ± 0.007 µg GA/50 g and 31.81 ± 0.005 µg Cf/50 g, with the maximum value being achieved after 30 min at 60 °C and 320 W, whereas the highest extraction values for TTC from the LP stem were 60.11 ± 0.003 µg TA/50 g, with the maximum value being achieved after 30 min at 40 °C and 320 W. With a nominal power of 320 W, the highest concentration is anticipated to occur between 30 min and 40 to 60 °C, with time and temperature having an indirect inversely proportional relationship to extraction. The maximal experimental value for total yield was found to be 22.81% after 30 min at 40 °C and 320 W. Hence, the time, water temperature, and nominal power all had an impact on the extraction. The model equation that represents the extraction of these metabolites using NCUSAE is shown in Equations (4)–(6), and the related modelled response surface is shown in Figure 5:

TAC (mg Cf/g) = −222.631 + (−0.591) × W + 8.501 × T + 3.694 × t + 0.001 × W^2^ + (−0.076 × T)^2^ + (−0.019 × t)^2^ + 0.002 × W × T + (−0.004) × W × t + (−0.021) × T × t (4)

TTC (mg TA/g) = −633.602 + 0.286 × W + 26.414 × T + 0.172 × t + 0.001 × W^2^ + (−0.249 × T)^2^ + (0.026 × t)^2^ + (−0.004) × W × T + (−0.011) × W × t + (−0.013) × T × t (5)

TPC (mg GA/g) = −529.345 + (−1.550) × W + 19.928 × T + 10.227 × t + 0.004 × W^2^ + (−0.174 × T)^2^ + (−0.036 × t)^2^ + 0.010 × W × T + (−0.014) × W × t + (−0.081) × T × t (6)

where W is nominal power, t is time (min), and T is temperature (°C).

### 3.9. Analytical Methods

#### 3.9.1. FT-IR

**Figure 6:**
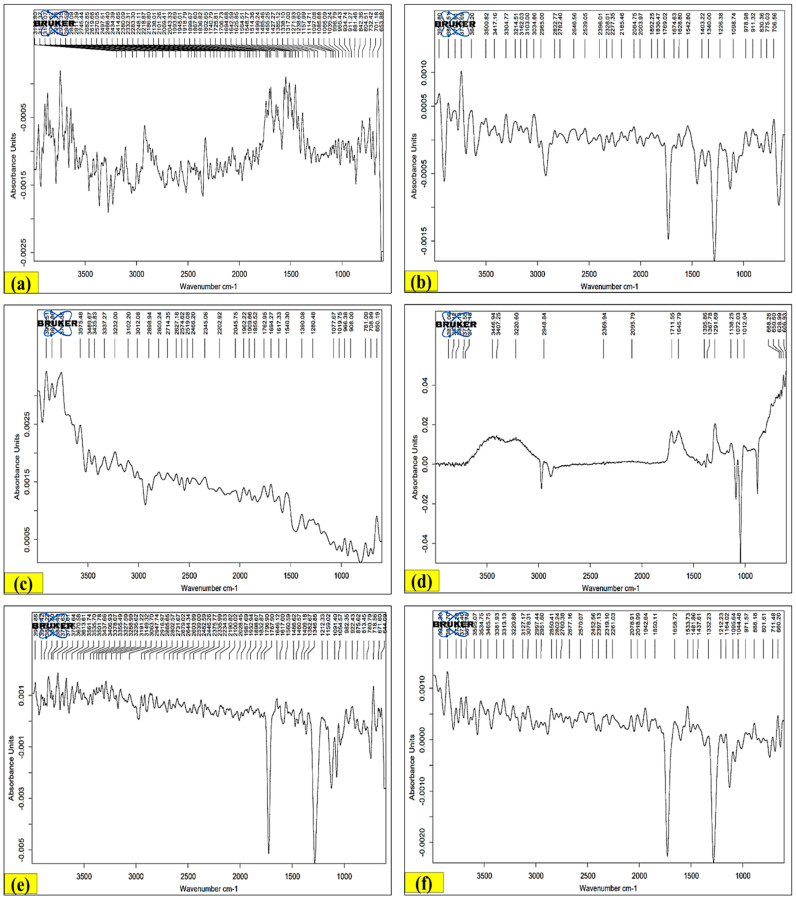

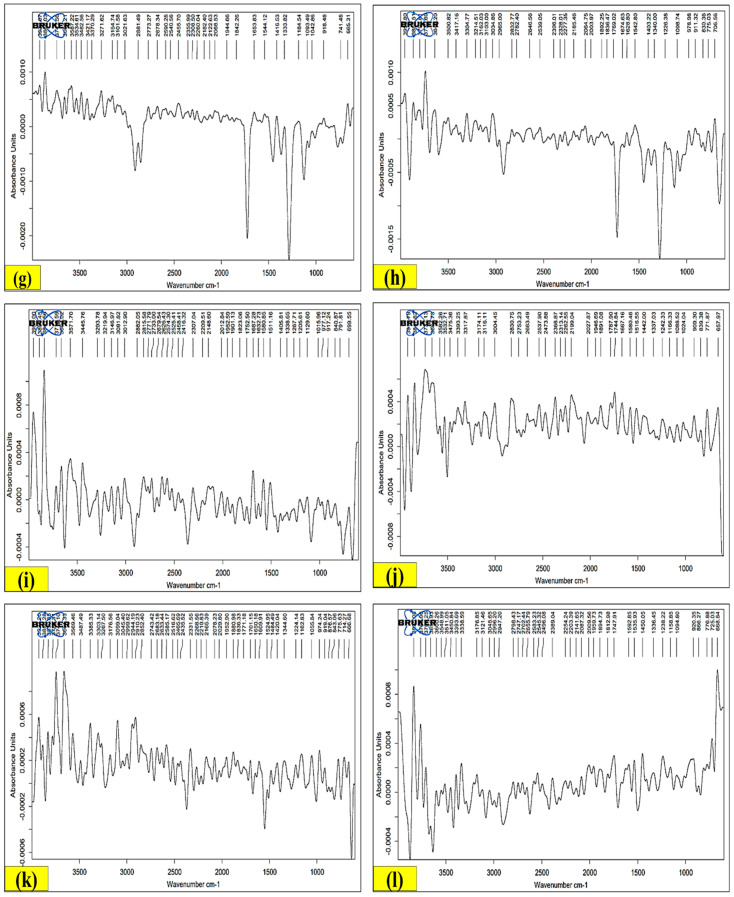
FT-IR spectra of all LP extracts: (**a**) LPSPE, (**b**) LPSH, (**c**) LPSCH, (**d**) LPSEA, (**e**) LPSE, (**f**) LPSAQ, (**g**) LPRPE, (**h**) LPRH, (**i**) LPRCH, (**j**) LPREA, (**k**) LPSE, (**l**) LPSAQ extracts.

The FTIR profile showed different characteristic peaks that were specifically ascribed to the presence of distinct functional groups or phytochemical compounds i.e., a band which occurred at 1625.85 cm^−1^ might be attributed to the presence of dienes (C=C); a band which occurred at 1035.34 cm^−1^ could be attributed to the presence of fluoroalkanes (C-X); a band which occurred at 881.46 cm^−1^ could be attributed to the presence of aromatic part (meta-substituted benzene) (C=H); a band which occurred at 701.18 cm^−1^ could be attributed to the presence of chloroalkanes (C-X); a band which occurred at 1322.68 cm^−1^ could be attributed to the presence of alcohols, carboxylic acids, esters, and ethers (C-O); a band which occurred at 1159.64 cm^−1^ could be attributed to the presence of tertiary alcohol (C-O); a band which occurred at 710.25 cm^−1^ could be attributed to the presence of an aromatic ring (Monosubstituted Benzene) (C=H); a band which occurred at 1427.58 cm^−1^ could be attributed to the presence of organophosphorus compound (aromatic) (P-C); a band which occurred at 805.23 cm^−1^ could be attributed to the presence of trisubstituted alkenes (C-H); a band which occurred at 1725.29 cm^−1^ could be attributed to the presence of aldehydes (C=O); a band which occurred at 1274.48 cm^−1^ could be attributed to the presence of carboxylic acids (C-O); a band which occurred at 1114.28 cm^−1^ could be attributed to the presence of esters (C-O); a band which occurred at 1066.08 cm^−1^ could be attributed to the presence of aliphatic amines (C-N); a band which occurred at 1767.90 cm^−1^ could be attributed to the presence of aliphatic alkynes (C≡C); a band which occurred at 1617.00 cm^−1^ could be attributed to the presence of primary amines (N-H); a band which occurred at 1091.40 cm^−1^ could be attributed to the presence of secondary alcohol (C-O); a band which occurred at 2903.37 cm^−1^ could be attributed to the presence of ammonium ions (N-H); a band which occurred at 1274.48 cm^−1^ could be attributed to the presence of phosphorus oxide (free) (P-O); and a band which occurred at 2069.79 cm^−1^ could be attributed to the presence of isothiocyanates (R-N=C=S) (C-N) (Figure 6) [65,66].

IR, MS, and ^1^H-NMR methods were used in India to identify extracted fatty sterols, acid alcohol, terpene, glycoside, hydrocarbon, and the associated compounds in the butanol and ethanol fractions of LP aerial parts [67,68]. In addition, IR was utilized to identify the amount of lipids in a petroleum ether extract of LP aerial parts in Egypt and to determine phytosterols from LP shoots in India [69,70]. The identification of distinguished fatty acids, alcohols, sterols, glycosides, hydrocarbons, terpenes, and their derivatives in the benzene and ethanol extracts of the aerial parts of LP was the result of further investigations carried out in India employing IR, ^1^H-NMR, and MS techniques. To evaluate the identification method, comparisons with real samples were also performed [67,68]. Additionally, in India and Egypt, the phytosterol content of the LP shoots was determined by IR analysis of the lipid content in the petroleum ether extract of the LP aerial parts [69,70].

#### 3.9.2. UV–Visible Profile

**Figure 7:**
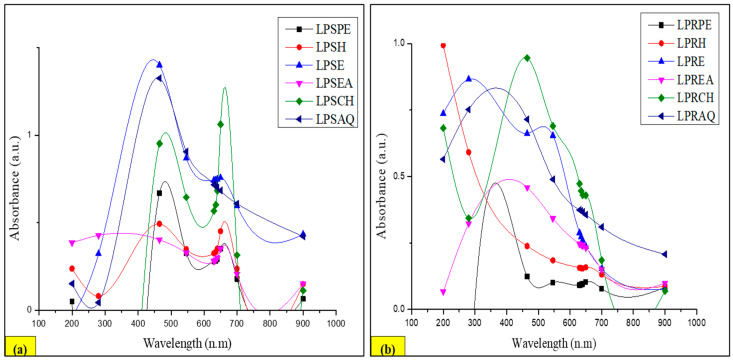
UV–visible spectra of both parts of LP: (**a**) stem, (**b**) root.

The UV–visible profile of the LP stem extracts revealed the presence of four peaks at ~465 nm for LPSPE, LPSH, LPSE, and LPSAQ, one peak at 650 nm for LPSCH, and one peak at 280 nm for LPSEA, with absorption ranges from 0 to 1.5 a.u. (Figure 7a). In contrast, the UV–visible profile of the LP root extracts revealed the presence of three peaks at ~465 nm for LPRPE, LPRCH, and LPREA, one peak at 546 nm for LPRH, and two peaks at 280 nm for LPRE and LPRAQ with absorption ranges from 0 to 1 a.u. (Figure 7b). In a study, a UV spectrophotometer was utilized to measure the TPC of ethanol crude extract of the aerial parts of LP, besides its ethyl acetate, butanol, and aqueous fractions, using the Folin–Ciocalteu method at 765 nm, and results were calculated in triplicate and expressed as mg of GA equivalent per g dry weight of plant material [51]. Moreover, it was also used to determine the sugar parts of flavonoids, as it was used in the structural elucidation process of six isolated flavonoids from the ethyl acetate fraction of LP aerial parts [71].

**Figure 8:**
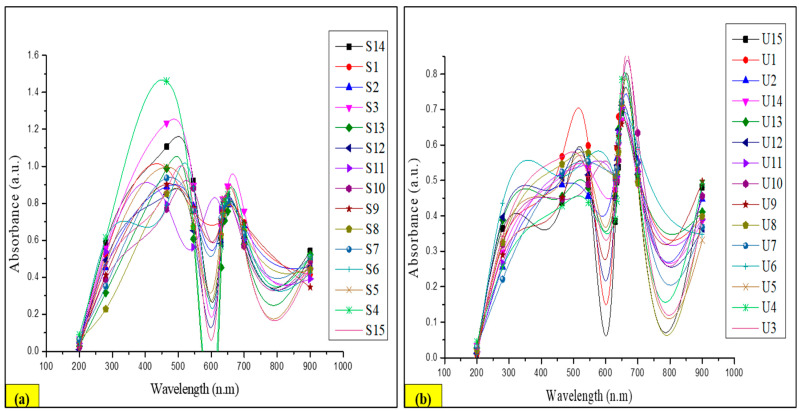
UV–visible spectra of both extraction methods of LP: (**a**) CSE extracts, (**b**) NCUSAE extracts.

The UV–visible profile of CSE extracts showed the existence of 15 peaks at 465 nm of S1 to S15 with absorption ranges from 0.00 to 1.46 a.u. (Figure 8a), while the UV–visible profile of NCUSAE extracts showed the presence of 15 peaks at 650 nm of U1 to U15 with absorption ranges from 0.00 to 0.78 a.u. (Figure 8b).

#### 3.9.3. Gas Chromatography–Mass Spectrometry (GC-MS)

**Figure 9:**
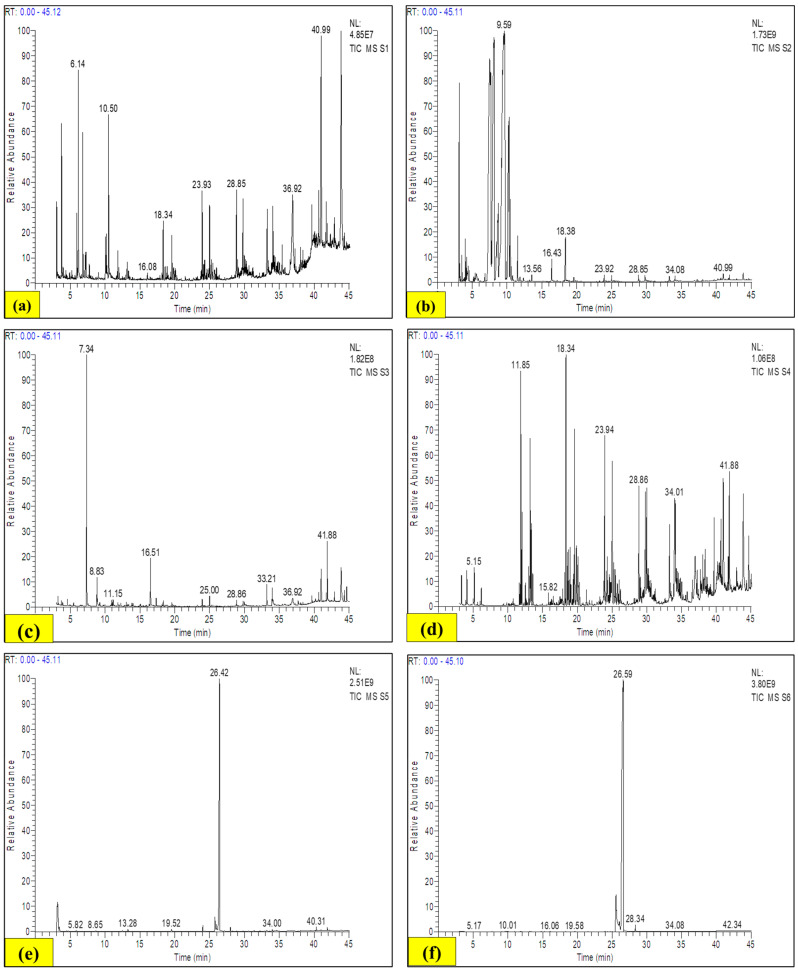

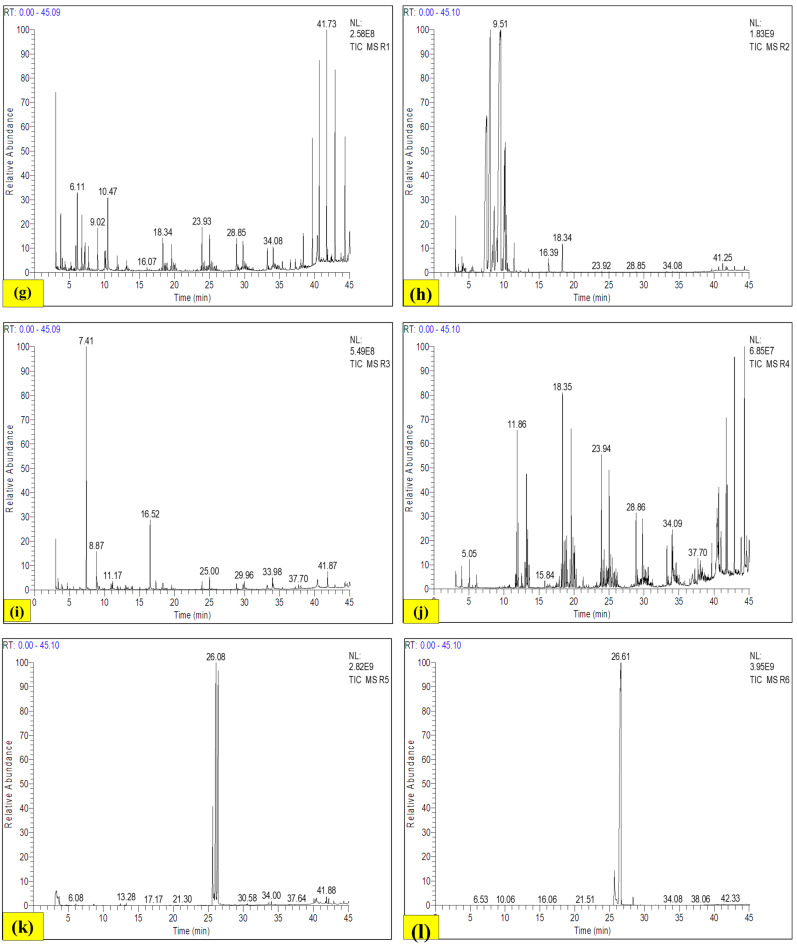
GC-MS spectra of all LP extracts: (**a**) LPSPE, (**b**) LPSH, (**c**) LPSCH, (**d**) LPSEA, (**e**) LPSE, (**f**) LPSAQ, (**g**) LPRPE, (**h**) LPRH, (**i**) LPRCH, (**j**) LPREA, (**k**) LPSE, (**l**) LPSAQ extracts.

In the present study, different extracts of LP stem and root were used for GC-MS analysis. The GC-MS result of the LP stem extracts disclosed about 66 main biologically active compounds (Table 2), whereas the LP root extracts showed the occurrence of 61 main biologically active components (Table 3). In the case of different LP stem extracts, 7,9-Di-tert-butyl-1-oxaspiro (4.5%), deca-6,9-diene-2,8-dione (1.13%), Hexadecane, 2,6,10,14-tetramethyl (3.07%), Ursolic aldehyde (1.13%), 25-Norisopropyl-9,19-cyclolanostan-2 2-en-24-one, 3-acetoxy-24-phenyl-4,4,14-trimethyl- (1.33%), 2,4-Hexanedione, 5,5-dimethyl- (4.44%), Hexanoic acid, 2-oxo-, methyl ester (3.48%), 1,5-Heptadien-4-one, 3,3,6-trimethyl- (28.20%), 2-Pentene, 4,4-dimethyl-, (E)- (5.52%), Carbamic acid, methyl ester (1.28%), n-Hexadecanoic acid (4.28%), 2,4-Di-tert-butylphenol (1.69%), Octadecanoic acid, 2,3-dihydroxypropyl ester (3.56%), and Myristic acid (4.90%) were the phytocompounds present in considerable amounts, and other chemicals were observed in very low amounts. In the case of distinct LP root extracts, Eicosane (5.18%), Oxalic acid, cyclohexyl pentyl ester (40.90%), 2-Propanone, 1,1-dichloro- (1.16%), Acetyl chloride, dichloro- (1.59%), Phenol, 4-chloro-2,6-bis(1,1-dimethylethyl)- (2.45%), Tetradecanoic acid (2.13%), n-Hexadecanoic acid (2.81%), l-(+)-Ascorbic acid 2,6-dihexadecanoate (2.18%), Hexadecanoic acid, 2-hydroxy-1-(hydroxymethyl)ethyl ester (3.69%), Glycerol 1-palmitate (3.69%), Octadecanoic acid, 2,3-dihydroxypropyl ester (2.93%), Octadecanoic acid, 2-hydroxy-1,3-propanediyl ester (2.93%), Hexadecane, 2,6,11,15-tetramethyl (4.63%), Hexadecanoic acid, and 1-(hydroxymethyl)-1,2-ethanediyl ester (3.69%) were the phytocompounds present in considerable amounts, and other compounds were found in lower amounts (Figure 9).

A study carried out on the petroleum ether extracts of LP whole plants cultivated in Qatar identified nine phytocompounds as myristic, lauric, palmitic, linoleic, palmitoleic, alpha-linolenic, oleic, behenic, and arachidic acids [40]. On the other hand, the hexane extract of the LP shoot cultivated in the UAE was found to include linoleic, heptadecanoic, α-linolenic, and n-hexadecanoic acids. In the UAE and Egypt [52], GC-MS analysis was utilized to study the fatty acids and their (methyl and ethyl ester) derivatives in the petroleum ether and hexane extracts of the LP aerial parts [67]. In another study, Campesterol was identified in the hexane extract and non-saponifiable material generated from acetone-soluble portions of petroleum ether extract of LP aerial parts in Egypt, the UAE, and India. In India, Stigmasterol was identified as well as isolated from the LP shoots. Additionally, it was discovered in the hexane and petroleum ether extracts of the LP shoots (Egypt and UAE), respectively [69,70,72].

There were 34 terpene compounds reported in the whole plant, aerial parts, leaves, and stems of LP. In an Indian study, Lupeol was found and measured in the methanol extract of the whole LP plant [73]. In various investigations carried out in India, Pakistan, Egypt, and the UAE, phenolic acids, flavonoids, phenolic aldehydes, coumarins, benzenediol, and phenylpropene were discovered to a large extent in the LP shoots. The methanol extract of the LP shoots included the following acids that were recognized and measured: vanillic acid (0.018%), caffeic acid (3.3%), ferulic acid, p-coumaric acid, cinnamic acid, and veratric acid [74]. The alkaloid portion of the methanol (defatted) extract of the LP aerial parts was analysed using GC-MS, and a total of 24 alkaloids, containing derivatives of pyridine, indole, pyrazine, and pyrrole, were discovered along with 6 other basic amines, notably N-ethyl-N-hydroxyethanamine, 4-aminobenzene-1,3-diol, 2-methylazetidine, N, N′-diphenylcarbodiimide, N-(prop-1-yn-1yl) acetamide, and 1,2-dimethylazetidine [71]. A study reported that the LP stems are a good source of peptides and amino acids. A study was carried out in India that showed the presence of two dipeptides, namely, glycyl-L-alanine and DL-alanyl-L-alanine, as well as six amino acids, namely, L-arginine, L-alanine, L-lysine, L-isoleucine, L-threonine, and L-methionine, in the LP stem by utilizing the GC-MS analytical tool [75].

One more study conducted in the UAE adopted the GC-MS method to examine the hexane extract of LP aerial parts, which led to the discovery of hydrocarbons, sterols, fatty acids, terpenes, and their derivatives [52]. In another investigation, GC-MS was used to determine the qualitative and quantitative amounts of phytosterols in LP shoots in India [70].

## 4. Conclusions

Biologically active compounds from conventional medicines have recently been used successfully as a targeted therapy to control a variety of health conditions. The results of proximate, qualitative, quantitative, and in vitro antioxidant analysis have made it clear that several phytocompounds (like phenols, saponins, tannins, flavonoids, and alkaloids) present in good amounts within different LP extracts have strong antioxidant efficacy and therapeutic applications, and can be utilized ethnobotanically to treat various illnesses. Different analytics were used, such as AAS for determining elemental composition and FT-IR and GC-MS for the identification of different phytocompounds present in LP that can be relevantly used in pharmaco-medical and agro-food industries. The findings of this research indicated that LPSPE and LPSE extracts of LP might become a source for new pharmaceuticals with potent antioxidant properties. This novel study also reveals the viability of phenol, tannin, and alkaloid extraction from LP stems using more environmentally friendly extraction methods based on the use of ethanol as a sustainable solvent. In terms of the extraction yield of all three biological chemicals, NCUSAE has been proven to be significantly more effective than CSE at a far lower energy cost, as well as being less time-consuming. The evaluated extraction processes were also effectively modelled using surface analysis, which provides an estimation of the best extraction under various experimental settings. To further establish the scientific perspective, our findings strongly recommend that the isolation of a particular phytocompound from the LP extracts (specifically LPSE extract) could be utilized prominently in drug discovery studies.

## Figures and Tables

**Table 1:** Analysis of variance for the determination of all models of CSE and NCUSAE.

	CSE				NCUSAE			
Sources of Variation	TPC	TAC	TTC	% Yield	TPC	TAC	TTC	% Yield
R	99.90%	91.33%	96.24%	99.25%	98.72%	95.15%	99.79%	99.04%
Predicted R	99.29%	0.00%	67.22%	92.07%	70.89%	0.00%	97.04%	86.88%
Adjusted R	99.73%	75.73%	89.47%	97.91%	96.40%	86.42%	99.41%	97.32%
PRESS	103.409	3814.42	2330.39	25.1612	3582.92	1782.86	129.972	41.0171
S	1.68121	5.92274	7.31087	0.688518	5.62258	3.98130	1.35556	0.773193

**Table 2:** GC-MS analysis of LP stem extracts.

S. No.	Phytochemical Compounds	RT (min)	Molecular Formulae	Molecular Weight	Peak Area (%)						Category
					LPSPE	LPSH	LPSCH	LPSEA	LPSE	LPSAQ	
1.	1-Propanol, 2-(2-hydroxypropoxy)-	10.19	C_6_H_14_O_3_	134	1.72	-	-	-	-	-	Glycol ether
2.	Hexadecane, 2,6,10,14-tetramethyl	25.00	C_20_H_42_	282	3.07	-	-	-	-	-	Phytane (diterpenoid hydrocarbon)
3.	Heptacosane	23.93	C_27_H_56_	380	3.33	0.14	0.97	4.35	0.14	-	Alkane
4.	Heneicosane	28.85	C_21_H_44_	296	3.47	-	1.13	4.35	-	-	Alkane
5.	7,9-Di-tert-butyl-1-oxaspiro(4,5)deca-6,9-diene-2,8-dione	33.21	C_17_H_24_O_3_	276	1.13	-	-	-	-	-	Flavonoid
6.	Ursolic aldehyde	33.21	C_30_H_48_O_2_	440	1.13	-	-	-	-	-	Triterpenoid
7.	Propanoic acid, 2-methyl-, (dodecahydro-6a-hydroxy-9a-methyl-3-methylene-2,9-dioxoazuleno[4,5-b]fu ran-6-yl)methyl ester	33.21	C_19_H_26_O_6_	350	1.13	-	-	-	-	-	Sesquiterpenoid
8.	Dihydromorphine, 2TMS derivative	39.78	C_23_H_37_NO_3_Si_2_	431	1.00	-	-	-	-	-	Morphinane alkaloid
9.	à-D-Glucopyranosiduronic acid, 3-(5-ethylhexahydro-2,4,6-trioxo-5-py rimidinyl)-1,1-dimethylpropyl 2,3,4-tris-O-(trimethylsilyl)-, methyl ester	39.78	C_27_H_52_N_2_O_10_Si_3_	648	1.00	-	-	-	-	-	Alpha-D-glucuronic acid (Steroid)
10.	1,2-Propanediol, 3-(hexadecyloxy)-, diacetate	39.78	C_23_H_44_O_5_	400	1.00	-	-	-	-	-	Phenol
11.	25-Norisopropyl-9,19-cyclolanostan-2 2-en-24-one, 3-acetoxy-24-phenyl-4,4,14-trimethyl-	39.89	C_35_H_48_O_3_	516	1.33	-	-	-	-	-	Steroid
12.	2,2,4-Trimethyl-3-pentanol	3.53	C_8_H_18_O	130	-	0.38	-	-	-	-	Terpenoid
13.	Cyclohexane, 1,4-dimethyl-	4.03	C_8_H_16_	112	-	0.62	-	-	-	-	Cycloalkane
14.	2-Octanol	4.42	C_8_H_18_O	130	-	0.45	-	-	-	-	Fatty alcohol
15.	3-Hexene, 2-methyl-, (Z)-	5.62	C_7_H_14_	98	-	0.33	-	-	-	-	Terpenoid
16.	3-Methyl-3-hexene	5.62	C_7_H_14_	98	-	0.33	-	-	-	-	Alkene
17.	2,4-Hexanedione, 5,5-dimethyl-	7.64	C_8_H_14_O_2_	142	-	4.44	-	-	-	-	Keto-enol tautomer of pivaloylacetone
18.	1-Butene-3-ethoxy	8.16	C_6_H_12_O	100	-	21.47	-	-	-	-	Dialkyl ether
19.	Hexanoic acid, 2-oxo-, methyl ester	8.75	C_7_H_12_O_3_	144	-	3.48	-	-	-	-	Oxo fatty acid
20.	Pentanoic acid, 2-propenyl ester	8.90	C_8_H_14_O_2_	142	-	0.20	-	-	-	-	Fatty acid
21.	Oxirane, [(hexyloxy)methyl]	9.20	C_9_H_18_O_2_	158	-	0.42	-	-	-	-	Hexyl glycidyl ether
22.	6,6-Dimethyl-1,3-heptadien-5-ol	9.20	C_9_H_16_O	140	-	0.42	-	-	-	-	Terpenoid
23.	3-Ethyl-3-methyl-2-pentanol	9.20	C_8_H_18_O	130	-	0.42	-	-	-	-	Terpenoid
24.	Hexane, 1,1′-oxybis	9.20	C_12_H_26_O	186	-	0.42	-	-	-	-	Ether
25.	1,5-Heptadien-4-one, 3,3,6-trimethyl-	9.69	C_10_H_16_O	152	-	28.20	-	-	-	-	Terpenoid
26.	Butane, 2,2,3,3-tetramethyl-	9.89	C_8_H18	144	-	0.22	-	-	-	-	Terpenoid
27.	Cyclopropane, 1,1,2,2-tetramethyl-	10.23	C_7_H_14_	98	-	5.52	-	-	-	-	Terpenoid
28.	1-Butene, 2,3,3-trimethyl-	10.23	C_7_H_14_	98	-	5.52	-	-	-	-	Terpene
29.	2-Pentene, 4,4-dimethyl-, (E)-	10.23	C_7_H_14_	98	-	5.52	-	-	-	-	Acyclic olefin
30.	Oxalic acid, cyclohexyl pentyl ester	10.23	C_13_H_22_O_4_	242	-	5.52	-	-	-	-	Ester of oxalic acid
31.	1-Hexanol, 2,2-dimethyl-	10.59	C_8_H_18_O	130	-	0.16	-	-	-	-	Tertiary alcohol
32.	3-Heptanone, 5-methyl-	10.59	C_8_H_16_O	128	-	0.16	-	-	-	-	Ketone
33.	N-Formylglycine	18.37	C_3_H_5_NO_3_	103	-	1.28	-	-	-	-	Carbohydrate derivative
34.	Carbamic acid, methyl ester	18.37	C_2_H_5_NO_2_	75	-	1.28	-	-	-	-	Ester of Carbamic acid
35.	Hexadecane, 2,6,11,15-tetramethyl	28.86	C_20_H_42_	282	-	-	0.97	5.51	-	-	Isoprenoid hydrocarbon
36.	Tetradecanoic acid	29.94	C_14_H_28_O_2_	228	-	-	1.17	4.90	-	-	Saturated fatty acid
37.	n-Hexadecanoic acid	33.98	C_16_H_32_O_2_	256	-	-	2.96	4.28	-	-	Palmitic acid
38.	Octadecanoic acid	37.71	C_18_H_36_O_2_	284	-	-	0.93	-	-	-	Saturated fatty acid
39.	4,4,5,7,8-Pentamethyl-6-chromanol	33.21	C_14_H_20_O_2_	220	-	-	3.59	-	-	-	Vitamin E
40.	Benzo[e]isobenzofuran-1,4-dione,1,3,4,5,5a,6,7,8,9,9a-decahydro-6,6,9a-trimethyl	33.21	C_15_H_20_O_3_	248	-	-	3.59	-	-	-	Terpenoid
41.	l-(+)-Ascorbic acid 2,6-dihexadecanoate	33.98	C_38_H_68_O_8_	652	-	-	2.96	4.28	-	-	Vitamin C
42.	10-Acetoxy-2-hydroxy-1,2,6a,6b,9,9,1 2a-heptamethyl-1,3,4,5,6,6a,6b,7,8,8a, 9,10,11,12,12a,12b,13,14b-octadecahy dro-2H-picene-4a-carboxylic acid,	40.19	C_33_H_52_O5	528	-	-	0.62	-	-	-	Terpenoid
43.	2,4-Di-tert-butylphenol	24.31	C_14_H_22_O	206	-	-	-	1.69	0.15	-	Phenol
44.	Phenol, 3,5-bis(1,1-dimethylethyl)-	24.31	C_14_H_22_O	206	-	-	-	1.69	0.15	-	Phenol
45.	2,6,10-Trimethyltridecane	25.46	C_16_H_34_	226	-	-	-	1.55	-	-	Terpenoid
46.	Tridecane, 2-methyl-	25.46	C_14_H_30_	198	-	-	-	1.55	-	-	Terpenoid
47.	Hexadecanoic acid, 2-hydroxy-1-(hydroxymethyl)ethyl ester	41.88	C_19_H_38_O_4_	330	-	-	-	3.56	-	-	Fatty acid ester
48.	Glycerol 1-palmitate	41.88	C_19_H_38_O_4_	330	-	-	-	3.56	-	-	Monoacylglycerols
49.	Octadecanoic acid, 2,3-dihydroxypropyl ester	41.88	C_21_H_42_O_4_	358	-	-	-	3.56	-	-	Stearic acid
50.	Benzoic acid, 2-benzoyl	27.12	C_14_H_10_O_3_	226	-	-	-	-	0.10	-	Aromatic carboxylic acid
51.	Myristic acid	29.99	C_14_H_28_O_2_	228	-	-	-	-	4.90	-	Fatty acid
52.	Phosphonoacetic Acid, 3TMS derivative	19.52	C_11_H_29_O_5_Psi_3_	356	-	-	-	-	0.83	-	Phosphonic acid
53.	1,1-Diphenyl-2-phenylthiobut-3-en-1-ol	27.12	C_22_H_20_OS	332	-	-	-	-	0.10	-	Thioether
54.	Benzophenone	27.12	C_13_H_10_O	182	-	-	-	-	0.10	-	Flavonoid
55.	Propanoic acid, 2-methyl-, (dodecahydro-6a-hydroxy-9a-methyl-3-methylene-2,9-dioxoazuleno[4,5-b] furan-6-yl) methyl ester, [3aS-(3aà,6á,6aà,9aá,9bà)]-	33.20	C_19_H_26_O_6_	350	-	-	-	-	0.22	-	Terpenoid
56.	1,2-Benzenedicarboxylic acid, butyl octyl ester	34.00	C_20_H_30_O_4_	334	-	-	-	-	0.33	-	Phthalic acid ester
57.	Erucic acid	34.58	C_22_H_42_O_2_	338	-	-	-	-	0.22	-	Fatty acid
58.	18,19-Secoyohimban-19-oic acid, 16,17,20,21-tetradehydro-16-(hydroxy methyl)-, methyl ester, (15á,16E)-	34.58	C_21_H_24_N_2_O_3_	352	-	-	-	-	0.22	-	Alkaloid (Yohimbine, an indole alkaloid)
59.	13-Docosenamide, (Z)	34.58	C_22_H_43_NO	337	-	-	-	-	0.22	-	Fatty acid amide
60.	Oxalic acid, mono-(5-[(2-bromophenyl)(2,2-dimeth ylpropionyloxy)methyl]-7,8-dihydro-5 H-[1,3]dioxolo[4,5-g]isoquinolin-6-yl) ester	42.20	C_24_H_26_BrNO_8_	535	-	-	-	-	0.14	-	Dicarboxylic acid
61.	5-Benzofuranacetic acid, 6-ethenyl-2,4,5,6,7,7a-hexahydro-3,6-d imethyl-à-methylene-2-oxo-, methyl ester	42.20	C_16_H_20_O_4_	276	-	-	-	-	0.14	-	Flavonoid
62.	2,6-Dihydroxybenzoic acid, 3TMS derivative	14.80	C_16_H_30_O_4_Si_3_	370	-	-	-	-	-	0.01	Hydroxybenzoic acid
63.	2,6-Dihydroxyacetophenone, 2TMS derivative	24.01	C_14_H_24_O_3_Si_2_	296	-	-	-	-	-	0.01	Phenol
64.	3,3a-Epoxydicyclopenta[a,d]cyclooctan-4á-ol, 9,10a-dimethyl-6-methylene-3á-isopropyl-	33.22	C_20_H_32_O_2_	304	-	-	-	-	-	0.01	Sesquiterpene
65.	25-Norisopropyl-9,19-cyclolanostan-22-en-24-one, 3-acetoxy-24-phenyl-4,4,14-trimethyl-	40.15	C_35_H_48_O_3_	516	-	-	-	-	-	0.06	Triterpenoid
66.	24,25-Dihydroxycholecalciferol	40.15	C_27_H_44_O_3_	416	-	-	-	-	-	0.06	Vitamin D

**Table 3:** GC-MS analysis of LP root extracts.

S. No.	Phytochemical Compound	RT (min)	Molecular Formulae	Molecular Weight	Peak Area (%)						Category
					LPRPE	LPRH	LPRCH	LPREA	LPRE	LPRAQ	
1.	1,5-Heptadien-4-one, 3,3,6-trimethyl-	9.02	C_10_H_16_O	152	2.18	-	-	-	-	-	Ketone
2.	Hexadecane, 2,6,10,14-tetramethyl-	33.26	C_20_H_42_	282	1.74	-	1.12	3.02	-	-	Phytane
3.	Tetratetracontane	34.08	C_44_H_90_	618	1.44	-	0.88	-	-	-	Alkane
4.	Eicosane	39.68	C_20_H_42_	282	5.18	-	-	2.90	-	-	Alkane
5.	Heptacosane	28.85	C_21_H_44_	296	2.16	-	0.87	-	0.31	-	Alkane
6.	2-Hexanone	4.18	C_6_H_12_O	100	-	0.26	-	-	-	-	Ketone
7.	(R)-(+)-3-Methylcyclopentanone 8	5.52	C_6_H_10_O	98	-	0.24	-	-	-	-	Cyclic ketone
8.	Oxalic acid, cyclohexyl pentyl ester	9.51	C_13_H_22_O_4_	242	-	40.90	-	-	-	-	Ester of oxalic acid and cyclohexyl pentanol
9.	2,2,4-Trimethyl-3-pentanol	3.48	C_8_H_18_O	130	-	0.20	-	-	-	-	Phenol
10.	2-Hexanol, 2,5-dimethyl-, (S)-	13.51	C_8_H_18_O	130	-	0.10	-	-	-	-	Terpenoid
11.	2-Heptanol, 2-methyl-	13.51	C_8_H_18_O	130	-	0.10	-	-	-	-	Alcohol
12.	3-Allyloxy-1,2 propanediol	16.38	C_6_H_12_O_3_	132	-	0.47	-	-	-	-	Glycerol 1-allyl ether
13.	1,2,3-Butanetriol	16.38	C_4_H_10_O_3_	106	-	0.47	-	-	-	-	Polyol
14.	Heptacosane	40.66	C_27_H_56_	380	-	0.08	0.70	2.68	0.76	0.02	Alkane
15.	Tetratetracontane	40.66	C_44_H_90_	618	-	0.08	1.33	4.00	0.76	-	Hydrocarbon compound
16.	N-Formylglycine	18.34	C_3_H_5_NO_3_	103	-	0.93	-	-	-	-	Carbohydrate derivative
17.	(2,3-Diphenylcyclopropyl) methyl phenyl sulfoxide, trans	41.24	C_22_H_20_OS	332	-	0.23	-	-	-	-	Sulfoxide
18.	3-Benzyl-2-phenyl-2,3,4,5-tetrahydro-1H-benzo[d]azepin	41.24	C_23_H_23_N	313	-	0.23	-	-	-	-	Benzodiazepine derivative
19.	Carbamic acid, methyl ester	18.34	C_2_H_5_NO_2_	75	-	0.93	-	-	-	-	Ester of Carbamic acid
20.	1,1′,1″-[5-methyl-1-pentene-1,3,5-triyl] tris-	41.24	C_24_H_24_	312	-	0.23	-	-	-	-	Terpenoids
21.	2-Propanone, 1,1-dichloro-	3.38	C_3_H_4_Cl_2_O	126	-	-	1.16	-	-	-	Dichloroacetone
22.	Propane, 1,1,3,3-tetrachloro-2-methyl-5	4.69	C_4_H_6_Cl_4_	194	-	-	0.90	-	-	-	Chloroalkane
23.	Acetyl chloride, dichloro-	11.16	C_2_HCl_3_O	146	-	-	1.59	-	-	-	Acetic acid
24.	Phenol, 4-chloro-2,6-bis(1,1-dimethylethyl)-	25.00	C_14_H_21_ClO	240	-	-	2.45	-	-	-	Phenol
25.	Tetradecanoic acid	29.96	C_14_H_28_O_2_	228	-	-	2.13	-	-	-	Fatty acid
26.	n-Hexadecanoic acid	33.99	C_16_H_32_O_2_	256	-	-	2.81	2.14	-	-	Palmitic acid
27.	l-(+)-Ascorbic acid 2,6-dihexadecanoate	33.99	C_38_H_68_O_8_	652	-	-	2.81	2.14	-	-	Vitamin C
28.	Octadecanoic acid	37.70	C_18_H_36_O_2_	284	-	-	1.04	-	-	-	Stearic acid
29.	Hexadecanoic acid, 2-hydroxy-1-(hydroxymethyl)ethyl ester	41.87	C_19_H_38_O_4_	330	-	-	2.93	3.69	-	-	Fatty acid ether
30.	Glycerol 1-palmitate	41.87	C_19_H_38_O_4_	330	-	-	2.93	3.69	-	-	Monoacylglycerol
31.	Octadecanoic acid, 2,3-dihydroxypropyl ester	41.87	C_21_H_24_O_4_	358	-	-	2.93	-	-	-	Glycerol ester of stearic acid
32.	Octadecanoic acid, 2-hydroxy-1,3-propanediyl ester	41.87	C_39_H_76_O_5_	624	-	-	2.93	-	-	-	Stearic acid
33.	Tetrapentacontane, 1,54-dibromo-	42.92	C_54_H_108_Br_2_	914	-	-	0.70	-	-	-	Alkane
34.	Octadecanoic acid, 2-hydroxy-1-(hydroxymethyl)ethyl ester	44.67	C_21_H_24_O_4_	358	-	-	0.96	1.63	-	-	Fatty acid ester
35.	Phenol, 3,5-bis(1,1-dimethylethyl)	24.31	C_14_H_22_O	206	-	-	-	1.58	-	-	Butylated hydroxytoluene (BHT)
36.	2,4-Di-tert-butylphenol	24.31	C_14_H_22_O	206	-	-	-	1.58	-	-	Phenol
37.	Hexadecane, 2,6,11,15-tetramethyl	25.01	C_20_H_42_	282	-	-	-	4.63	-	-	Crocetane
38.	Hexadecanoic acid, 1-(hydroxymethyl)-1,2-ethanediyl ester	41.88	C_35_H_68_O_5_	568	-	-	-	3.69	-	-	Palmitic acid
39.	Octadecanoic acid, 2-hydroxy-1-(hydroxymethyl)ethyl ester	44.68	C_21_H_42_O_4_	358	-	-	-	1.63	-	-	Fatty acid ester
40.	3-Buten-1-ol, TMS derivative	13.04	C_7_H_16_OSi	144	-	-	-	-	0.11	-	Homoallyl alcohol
41.	1,1-Diphenyl-2-phenylthiobut-3-en-1-ol	26.76	C_22_H_20_OS	332	-	-	-	-	0.15	-	Thioether (phenylpropanoid)
42.	Benzophenone	26.76	C_13_H_10_O	182	-	-	-	-	0.15	-	Flavonoid
43.	Benzoic acid, 2-benzoyl	26.76	C_14_H_10_O_3_	226	-	-	-	-	0.15	-	Aromatic carboxylic acid
44.	Heptacos-1-ene	30.48	C_27_H_54_	378	-	-	-	-	0.18	-	Alkene
45.	1-Hexadecanol, 2-methyl	30.48	C_17_H_36_O	256	-	-	-	-	0.18	-	Fatty alcohol
46.	1,2-Benzenedicarboxylic acid, butyl 8-methylnonyl ester	33.65	C_22_H_34_O_4_	362	-	-	-	-	0.28	0.01	Phthalate
47.	Phthalic acid, butyl nonyl ester	33.65	C_21_H_32_O_4_	348	-	-	-	-	0.28	-	Phthalic acid ester
48.	Tetrapentacontane, 1,54-dibromo-	40.65	C_54_H_108_Br_2_	914	-	-	-	-	0.16	-	Fatty acid
49.	Ethanol, 2-(octadecyloxy)	40.65	C_20_H_42_O_2_	314	-	-	-	-	0.16	-	Glycol ether
50.	1,2-Propanediol, 3-(octadecyloxy)-, diacetate	41.00	C_25_H_48_O_5_	428	-	-	-	-	0.42	0.02	Fatty acid ester
51.	Oxalic acid, mono-(5-[(2 bromophenyl) (2,2-dimeth yl propionyloxy) methyl]-7,8-dihydro-5 H-[1,3] dioxolo[4,5-g] isoquinolin-6-yl) ester	41.88	C_24_H_26_BrNO_8_	535	-	-	-	-	0.61	-	Dicarboxylic acid
52.	Oleic acid, 3-(octadecyloxy)propyl ester	43.90	C_39_H_76_O_3_	592	-	-	-	-	0.22	0.02	Fatty acid ester
53.	Ethanol, 2-(octadecyloxy)-6	28.85	C_20_H_42_O_2_	314	-	-	-	-	-	0.02	Fatty alcohol
54.	1,2-Benzenedicarboxylic acid, butyl 8-methylnonyl ester	32.16	C_22_H_34_O_4_	362	-	-	-	-	-	0.01	Phthalate
55.	7,9-Di-tert-butyl-1-oxaspiro (4,5) deca-6,9-diene-2,8-dione	33.20	C_17_H_24_O_3_	276	-	-	-	-	-	0.02	Flavonoid
56.	Propanoic acid, 2-methyl-, (dodecahydro-6a-hydroxy-9a-methyl-3-methylene-2,9-dioxoazuleno [4,5-b] furan-6-yl)methyl ester, [3aS-(3aà,6á,6aà,9aá,9bà)]-	33.20	C_19_H_26_O_6_	350	-	-	-	-	-	0.02	Sesquiterpenoid
57.	Betulinaldehyde	33.20	C_30_H_48_O_2_	440	-	-	-	-	-	0.02	Triterpenoid
58.	9,10-Secocholesta-5,7,10(19)-triene-3, 24,25-triol, (3á,5Z,7E)-	38.06	C_27_H_44_O_3_	416	-	-	-	-	-	0.02	Steroid
59.	24,25-Dihydroxycholecalciferol	38.06	C_27_H_44_O_3_	416	-	-	-	-	-	0.02	Vitamin D3 derivative
60.	Dodecyl cis-9,10-epoxyoctadecanoate	39.02	C_30_H_58_O_3_	466	-	-	-	-	-	0.01	Epoxy fatty acid
61.	Spirost-8-en-11-one, 3-hydroxy-, (3á,5à,14á,20á,22á,25R)-	40.61	C_27_H_40_O_4_	428	-	-	-	-	-	0.02	Steroid

## Data Availability

The datasets used and/or analysed during the current study are available from the corresponding author on reasonable request. The data also presented in this study are available in Supplementary Materials.

## Supplementary Materials

The following supporting information can be downloaded at: https://www.mdpi.com/article/10.3390/antiox13070794/s1, Table S1: Experimental design for conventional soxhlet extraction (CSE) and non-conventional ultrasound-assisted extraction (NCUSAE); Table S2: Qualitative screening of *Leptadenia pyrotechnica* (LP) stem and root extracts.

## Abbreviations

LP: *Leptadenia pyrotechnica* Forssk. Decne; AAS, atomic absorption spectrophotometer; UV–vis, ultraviolet–visible; FT-IR, Fourier-transform infrared spectroscopy; GC-MS, gas chromatography–mass spectrometry; CSE, conventional soxhlet extraction; NCUSAE, non-conventional ultrasound-assisted extraction; GA, gallic acid; LOD, loss of dryness; µg/mL, micrograms per milliliters; g, gram; nm, nanometers; CCD, central composite design; ANOVA, analysis of variance; RSREG, response surface regression; TAV, total ash value; WSA, water-soluble ash; AIA, acid-insoluble ash; TEAC, trolox-equivalent antioxidant capacity; DPPH, 2,2-diphenyl-1-picryl-hydrazyl-hydrate; MC, metal chelation; M, methanol; NO, nitric oxide; RT, room temperature; RSM, response surface methodology; TPC, total phenolic content; TSC, total saponin content; TTC, total tannin content; TFC, total flavonoid content; TAC, total alkaloid content; WHO, World Health Organization; AQ: aqueous; B, benzene; Bu, butanol; CH, chloroform; EA, ethyl acetate; E, ethanol; FRAP, ferric reducing antioxidant power assay; H, hexane; PE, petroleum ether; H_2_O_2_, hydrogen peroxide; S/M, solvent/material.
