# Supplementary material for: Qualitative, Quantitative, In Vitro Antioxidant Activity and Chemical Profiling of Leptadenia pyrotechnica (Forssk.) Decne Using Advanced Analytical Techniques
Source: Antioxidants (Basel). 2024 Jun 28;13(7):794. doi: 10.3390/antiox13070794 (PMC11273909; doi:10.3390/antiox13070794)
Supplement: Supplementary file 1 [file antioxidants-13-00794-s001.zip › antioxidants-3063119-supplementary.pdf]

Qualitative, quantitative, *in vitro* antioxidant activity, and chemical profiling of *Leptadenia pyrotechnica* (Forssk.) Decne using advanced analytical techniques

Divya Kumari[1], Devendra Singh[2], Mukesh Meena[3], Pracheta Janmeda[1,*], Manzer H. Siddiqui[4]

[1]Department of Bioscience and Biotechnology, Banasthali Vidyapith, Tonk 304022, Rajasthan, India; divyakumari4193@gmail.com (DK), pracheta@banasthali.in (PJ)

[2]Department of Chemistry, Mohanlal Sukhadia University, Udaipur 313001, Rajasthan, India; dsingh@mlsu.ac.in

[3]Laboratory of Phytopathology and Microbial Biotechnology, Department of Botany, Mohanlal Sukhadia University, Udaipur 313001, Rajasthan, India; mukeshmeenamlsu@gmail.com/ drmukeshmeena321@mlsu.ac.in

[4]Department of Botany and Microbiology, College of Science, King Saud University, Riyadh 11451, Saudi Arabia; mhsiddiqui@ksu.edu.sa

***Correspondence:**

Pracheta Janmeda: pracheta@banasthali.in

ORCID: https://orcid.org/0000-0003-0500-4636

Supplementary Table S1. Experimental design for conventional soxhlet extraction (CSE) and non-conventional ultrasound-assisted extraction (NCUSAE).

Extraction methods	Factors	Levels	Response variables
**CSE**	Solvent: material ratio (ml/g)	300/10; 350/10; 400/10	Extraction of alkaloids, tannin, phenols, and % yield
	Time (h)	18; 26; 34	
	Temperature (◦C)	40; 50; 60	
**NCUSAE**	Nominal power (W)	120; 220; 320	
	Temperature (◦C)	40; 50; 60	
	Time (min)	30; 45; 60	

Supplementary Table S2. Qualitative screening of *Leptadenia pyrotechnica* (LP) stem and root extracts.

**Plant constituents**	**Tests**	**Indication**	**Stem**						**Root**					
			**LPSPE**	**LPSH**	**LPSCH**	**LPSEA**	**LPSE**	**LPSAQ**	**LPRPE**	**LPRH**	**LPRCH**	**LPREA**	**LPRE**	**LPRAQ**
**Primary metabolites**														
Proteins and Amino acids	Biuret test	Peptide bond	-	-	-	-	-	-	-	-	-	-	-	-
	Ninhydrin test	Aliphatic primary amine and amino acids	-	-	-	-	-	-	-	-	-	-	-	-
	Xanthoprotein test	Tyrosine and tryptophan	-	-	-	-	-	-	-	+	-	-	-	-
	Millon Nasses test	Tyrosine and xanthoproteic acid	-	-	-	-	-	-	-	-	-	-	-	-
	Protein test	Protein containing sulphur	-	-	+++	-	+	-	-	-	+	-	-	-
	Tyrosine test	Tyrosine	-	-	-	-	-	-	-	-	-	-	-	-
	Cysteine test	Cysteine	-	-	+++	+	-	-	-	-	-	-	-	-
Carbohydrates	Fehling's test	Reducing sugar	-	-	-	-	-	-	-	-	-	-	-	-
	Iodine test	Starch, dextrin, and glycogen	-	-	-	-	-	-	-	-	-	-	-	-
	Cobalt chloride test	Glucose and fructose	+++	+++	+++	++	+	++	+++	+++	++	+++	+	+++
	Barfoed's test	Monosaccharides	-	-	-	-	+	-	-	-	-	-	-	-
	Seliwanoff's test	Ketohexoses and fructose	-	+	-	-	-	-	-	-	-	-	-	-
	Bial's orcinol test	Pentose and pentosans	-	-	-	-	-	-	-	-	-	-	-	-
	Tannic acid test	Starch	-	-	-	-	+++	+	-	-	-	+	++	++
Lipids	Saponification test	Oil and fat	-	-	-	-	+	+	+	+	+	+	+	+
**Secondary metabolites**														
Alkaloids	Dragendroff's reagent test	Secondary and tertiary amines	+	+	+++	++	++	-	+	+	++	+	+	-
	Murexide test	Purines	-	-	-	-	-	-	-	-	-	-	-	-
	Tannic acid test	Cinchonine, caffeine, strychnine, and quinine	-	+++	++	+	-	+	-	-	-	+++	+	++
Glycosides	Keller Killiani test	Cardiac glycosides, bufadienolide, and free sugar	+	++	-	-	+++	-	++	++	-	-	+	+
	Legal's test	Cardenolides	+	++	++	+	+	+	+	+	++	+++	++	++
	Borntrager's test	Anthraquinones	-	-	-	-	+	-	-	-	-	+	-	-
Flavonoids	Shinoda test	Xanthones, flavonones, and flavononols	-	-	-	-	+	-	-	-	-	-	-	-
	Sulphuric acid test	Chalcones, aurons, flavanes, flavones, and flavonols	++	-	-	+	+	-	-	-	+	+	+	+

Phenols	Potassium dichromate test	Aldehydes and ketones	+	+	-	++	+	+	+	+	-	+	+	++
	Lead acetate test	Phenolic compound	+++	+++	++	++	++	-	+++	++	+	++	-	-
Tannins	Ferric chloride ($FeCl_3$) test	Cathechic tannin	-	-	+++	+	+	-	-	-	-	-	-	-
	Bromine water	Condensed tannin	+	+	-	-	-	+	++	++	+	++	+	+
	Hydrochloric acid (HCl) test	Phlobatannins	-	+	-	-	-	+	-	-	+	+	+	-
Saponins	Foam test	Saponin	++	+	+	+	++	+	+	+	+	+	++	+
Terpenoids	Terpenoid test	Terpenoid	+++	+	-	++	+++	-	-	-	-	-	-	-
Steroids	Salkowski test	Phytosterols and cholesterol	+	-	+	-	-	-	-	-	-	-	-	-
Acidic compound	Sodium bicarbonate test		++	++	+++	++	++	+	-	+	+	++	+++	+++
Organic acids	$FeCl_3$ test	Malic acid	++	+	-	+	+	+	+	+	+	+	++	+
	Lead acetate test	Oxalic acid	+	++	+	+	+	+++	++	+	+	+	++	+++
	Tartaric acid test	Tartaric acid	-	-	+	-	-	-	-	-	-	-	-	-
	Citric acid test	Citric acid	-	-	++	+	++	-	-	-	+	-	-	+
Vitamins	Vitamin test	Vitamin C	-	-	+	-	+	-	-	-	+	-	-	-
Volatile oils	Solubility test	Volatile oils	-	-	+	++	+++	++	-	+	-	+	++	+

Note: - (absent), + (less quantity), ++ (moderate quantity), +++ (high quantity).
